# Embedding supervised exercise training for men on androgen deprivation therapy into standard prostate cancer care: a feasibility and acceptability study (the STAMINA trial)

**DOI:** 10.1038/s41598-021-91876-y

**Published:** 2021-06-14

**Authors:** Sophie Reale, Rebecca R. Turner, Eileen Sutton, Liz Steed, Stephanie J. C. Taylor, Dylan Morrissey, Patrick Doherty, Diana M. Greenfield, Michelle Collinson, Jenny Hewison, Janet Brown, Saïd Ibeggazene, Malcolm Mason, Derek J. Rosario, Liam Bourke

**Affiliations:** 1 Allied Health Professionals, Radiotherapy and Oncology, Sheffield Hallam University, Sheffield, UK; 2 Population Health Sciences, University of Bristol, Bristol, UK; 3 Institute for Population Health Sciences, Queen Mary University of London, London, UK; 4 Sports and Exercise Medicine, School of Medicine and Dentistry, William Harvey Research Institute, Queen Mary University of London, London, UK; 5 Department of Health Sciences, University of York, York, UK; 6 Specialised Cancer Services, Sheffield Teaching Hospital NHS Foundation Trust, Sheffield, UK; 7 Clinical Trials Research Unit, Leeds Institute of Clinical Trials Research, University of Leeds, Leeds, UK; 8 School of Medicine, Leeds Institute of Health Sciences, University of Leeds, Leeds, UK; 9 Department of Oncology and Metabolism, University of Sheffield, Sheffield, UK; 10 School of Medicine, Cardiff University, Cardiff, UK; 11 Department of Urology, Sheffield Teaching Hospitals, Sheffield, UK

**Keywords:** Psychology, Health care, Urology

## Abstract

Lifestyle interventions involving exercise training offset the adverse effects of androgen deprivation therapy in men with prostate cancer. Yet provision of integrated exercise pathways in cancer care is sparse. This study assessed the feasibility and acceptability of an embedded supervised exercise training intervention into standard prostate cancer care in a single-arm, multicentre prospective cohort study. Feasibility included recruitment, retention, adherence, fidelity and safety. Acceptability of behaviourally informed healthcare and exercise professional training was assessed qualitatively. Despite the imposition of lockdown for the COVID-19 pandemic, referral rates into and adherence to, the intervention was high. Of the 45 men eligible for participation, 79% (n = 36) received the intervention and 47% (n = 21) completed the intervention before a government mandated national lockdown was enforced in the United Kingdom. Patients completed a mean of 27 min of aerobic exercise per session (SD = 3.48), at 77% heart rate maximum (92% of target dose), and 3 sets of 10 reps of 3 resistance exercises twice weekly for 12 weeks, without serious adverse event. The intervention was delivered by 26 healthcare professionals and 16 exercise trainers with moderate to high fidelity, and the intervention was deemed highly acceptable to patients. The impact of societal changes due to the pandemic on the delivery of this face-to-face intervention remain uncertain but positive impacts of embedding exercise provision into prostate cancer care warrant long-term investigation.

## Introduction

Prostate cancer is common—responsible for a quarter of all new diagnoses of male cancers in the UK^1^. Around 50% of cases will be locally advanced or metastatic at presentation. Androgen deprivation therapy (ADT, medical castration), remains the cornerstone of therapy, combined with radiotherapy for locally advanced disease^2^, with taxane-based chemotherapy for metastatic disease^3^ or as sole treatment. ADT is effective for prostate cancer; the 10-year cancer-specific survival for locally advanced prostate cancer is around 55%. For those with metastatic prostate cancer, relative survival is however just 30% over 5 years^4^.

A recent systematic review assessing adverse effects across the ADT treatment spectrum (including impacts of Abiraterone Acetate and Enzalutamide) reported such treatment will significantly increase the risk of adverse effects related to musculoskeletal, metabolic, cardiac, nervous system, vascular, hepatobiliary and reproductive systems alongside psychiatric and general disorders^5^. Such wide-ranging adverse impacts on health inevitably translates to negative consequences for men’s quality of life (QoL)^6,7^.

Of the numerous strategies evaluated, exercise training is the only evidence-based intervention to have demonstrated beneficial effects exceeding clinically relevant thresholds for disease-specific QoL, cardiovascular health and other important patient reported outcomes such as cancer-specific fatigue^8,9^. Current National Institute of Health and Care Excellence guidelines recommend 12-weeks of combined resistance and aerobic exercise as standard treatment (NG 131 1.4.19) for men on ADT for prostate cancer^10^, based on findings from non-NHS (National Health Service) practice^8,11–13^. The current European Association of Urology guidelines make similar recommendations^14^. Nevertheless, there is very little evidence of such provision being part of standard cancer care or trial data demonstrating how to maintain long-term benefits^6^. A systematic review of randomised controlled trials (RCTs) demonstrated important initial effects with such a 12-week exercise programme, but the benefits dissipated without behavioural support, primarily instruction on how to perform the behaviour, with practise, and goal setting^15^. Behavioural support is an essential component of complex exercise interventions to facilitate uptake, adoption, maintenance and relapse management of exercise behaviour and maximise adherence to the prescribed dose^16^. Supervision is essential for safe translation of the reported efficacy via effectiveness trials to the wider patient population.

Despite clear national and international best practice clinical guidelines, referral and provision of integrated exercise pathways in cancer care is notably sparse and quality assurance is rarely a routine part of assessment^6^. A previous phase II trial has demonstrated how behaviour change techniques (BCTs) (i.e. goal setting, generalisation of target behaviour, prompting self-monitoring and prompting practise) can support inactive men with prostate cancer on active surveillance to improve and maintain regular exercise behaviour for up to 12 months of follow-up^17^. However, it is still not known how to embed such pathways into standard prostate cancer care or if clinically relevant outcomes can be achieved over long-term follow-up in men with advanced disease. In line with recent updated CONSORT guidance^18^ the purpose of this study was to assess the acceptability and feasibility of embedding a supervised exercise pathway into standard NHS prostate cancer care to meet current National Institute of Health and Care Excellence (NG131 1.4.19) best practice recommendations. Data collected here will be used to inform a planned definitive trial evaluating the clinical and cost-effectiveness of a tailored lifestyle intervention for men with prostate cancer on ADT.

## Methods

### Study design

The present study was a single-arm multicentre interventional cohort study (18/03/2019, ISRCTN15691664) designed to examine the acceptability and feasibility of integrating and delivering exercise into standard prostate cancer care, as part of our intervention development process. This study contributes to a larger body of work; Supported exercise TrAining for Men with prostate caNcer on Androgen deprivation therapy (STAMINA—a National Institute of Health Research funded programme grant for applied research). In STAMINA we propose to embed our behaviourally informed and evidence-based lifestyle intervention into prostate cancer care. This involves training cancer team members to endorse and support exercise, community-based exercise trainers (ETs) to deliver supervised exercise and behavioural support and communication between them to provide seamless patient care (see supplementary file 1).

### Participants

Participants included (1) men on ADT for the treatment of prostate cancer, (2) healthcare professionals (HCPs) involved in diagnosis and/ or treatment and/ or follow-up of men with prostate cancer on ADT and (3) community-based ETs. Inclusion and exclusion criteria can be seen in supplementary file 2.

### Procedures

#### Recruitment of participating services

NHS sites and a linked community exercise partner (https://www.nuffieldhealth.com) within ~ 16 km radius, were identified from a previously published national survey of prostate cancer care teams^6^ and chosen to reflect heterogeneity of acute trusts treating prostate cancer based on geographical location, prostate cancer management pathways and professional leads. Approvals were sought through NHS trusts’ clinical directorate routes, as well as applicable departmental and clinical governance approvals before inviting HCPs and ETs to participate. Regulatory and ethical approvals, in accordance with the Helsinki Declaration, were sought prior to the commencement of research activities (NHS REC reference: 19/NW/0025).

#### Recruitment of study participants

Men on ADT for prostate cancer were identified and recruited for exercise by STAMINA trained HCPs in secondary care outpatient clinics. All men on ADT were recommended exercise and referred to exercise (where eligible) as an essential part of prostate cancer care, by trained HCPs, at diagnosis and follow-up appointments. Trained HCPs addressed patient barriers and facilitators to exercise using theory and evidence-based BCTs (e.g. problem solving, pros and cons, verbal persuasion about capability) and provided men with study details and materials (behaviourally informed leaflet on potential adverse effects of ADT, benefits of exercise and lifestyle modification and information about the community exercise partner).

Eligible participants were referred to the research team for telephone consent and completion of a pre-exercise health screening questionnaire used in previous trials^17^ (adapted for men on ADT). On completion, a baseline assessment assessing weight, height, resting heart rate, blood pressure and functional capacity via chair sit to stand test^19^ was carried out in the clinic by a research nurse before referral to the linked community exercise partner. The assessments were repeated at 12-weeks.

#### The embedded training programme

##### Professional training

Eligible HCPs and ETs received behaviourally informed, face-to-face, practical skill-based training to: (1) improve knowledge (2) increase confidence, (3) change beliefs, (4) establish social norms, (5) develop behaviour change skills and (6) change beliefs of their perceived role to support patient exercise behaviour^20,21^. HCP training was delivered within 7 days prior to the NHS sites recruitment start date and lasted a duration of half a day. ET training was delivered within 7 days post recruitment start date to reduce time between training and delivery of the exercise prescription (further details available in supplementary file 1).

##### The patient intervention

Supervised aerobic and resistance training were undertaken twice weekly for 12 weeks at Nuffield Health as per National Institute of Health and Care Excellence recommendations (NG131 1.4.19). The exercise prescription was tailored to account for the individual’s capability, opportunity and motivation and (where appropriate) any relevant medications and co-morbidities using applicable clinical exercise prescription guidelines and results of a submaximal treadmill/ bike exercise test based on the modified Bruce protocol^22^.

According to our previously published data^17^, to facilitate optimum behaviour change, in those participants who need to overcome significant functional limitations due to comorbidity or lack of confidence in a public gym environment, we allocated a 'run in period' which is a ramped approach to working towards the full intervention dose. This was tailored to each individual's capability and is also an important safety aspect of our intervention design. These men were offered a reduced exercise prescription for (up to) the first 6-weeks, followed by 6 weeks according to protocol (12 weeks in total).

Supervised exercise sessions were delivered face-to-face by an ET, one-to-one or in small groups (maximum, n = 5), with behavioural support (i.e. instruction, demonstration, prompting practise, feedback on behaviour, graded task). Each session consisted of an exercise programme that has been previously shown to be well-tolerated and delivers clinically significant improvements in cancer specific QoL and fatigue levels^8^. Briefly this entailed: 30–45 min of moderate intensity aerobic exercise and up to four sets and 8–12 repetitions of resistance exercises targeting major muscle groups. Exercise intensity was set at between 65 and 85% of age predicted heart rate or 3 to 5 on the 10-point Borg rating of perceived exertion (RPE)^23^. Heart rate and RPE were measured at 5-min intervals and the exercise intensity amended where optimal heart rate was not achieved. Ongoing feedback on exercise technique and progressions were provided throughout the supervised sessions as appropriate.

**Figure 1:**
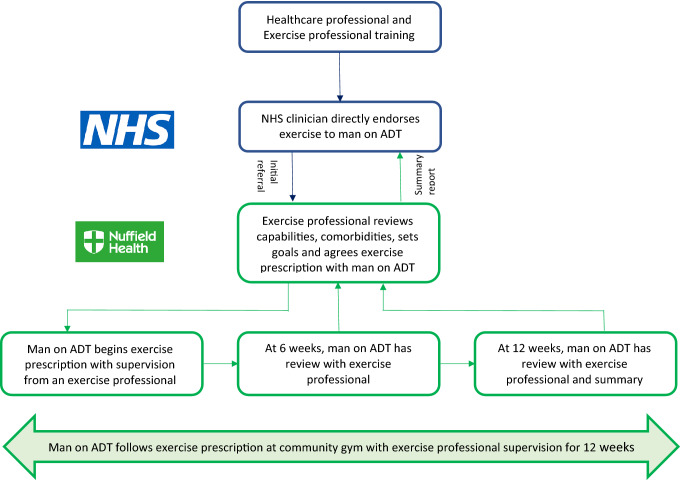
Overview of the study procedure.

Behavioural support was provided through an interactive patient workbook, ad hoc during supervised exercise sessions and more formally at 6 and 12 weeks in progress reviews (with ETs) and follow-up care (with HCP). The primary focus was on integrating exercise into weekly routine (week 6) and maintaining lifestyle modification (week 12) by utilising the following BCTs: feedback on behaviour, goal setting, review goals, prompt self-monitoring, discuss social support and discuss habit formation. At 12 weeks, the sub-maximal exercise test was repeated, and a summary report was produced by the ETs and sent to the HCP team for review with the patient as part of their standard follow-up care. Behaviour change support was informed by theory and evidence^24^, including evidence from a previously reported Cochrane review exploring interventions to improve exercise behaviour in people living with and beyond cancer^16^. A simplified overview of the procedure can be seen in Fig. 1.

### Outcome measures

#### Feasibility

Process data comprising recruitment; adherence to the intervention; study retention and adverse event rate were assessed by extracting data from screening and recruitment logs, attendance at supervised exercise sessions and exercise diary records, summary report completion and review of adverse event logs. Adverse events were defined according to international ethical, scientific and practical standards, i.e. Good Clinical Practice^25^.

#### Acceptability

Acceptability of the interventions were explored in semi-structured interviews with (1) men on ADT for prostate cancer (purposively sampled to include variation in age, ADT duration, functional capacity, previous exercise behaviour, ethnicity), (2) HCPs in the prostate cancer care pathway (purposively sampled to include different team members) and (3) community-based ETs (sampled to include both personal trainers, physiologists, general managers and fitness managers). Interviews were led by an experienced facilitator (ES, SR, RT) and the topic guide was reviewed by the dedicated STAMINA Patient and Public involvement (PPI) group. Interviews explored the seven component constructs of the Theoretical Framework of Acceptability (TFA;^26^). Interviews were conducted post-intervention face-to-face or via telephone, dependent on participant convenience, and were audio recorded. Please see supplementary file 3 for topic guides.

#### Fidelity

Where acceptable and possible, interactions between patients and HCPs, and patients and ETs, were digitally audio-recorded (during initial appointment, six- and twelve-week reviews) as a measure of treatment fidelity i.e. to examine the ability of HCPs and ETs to perform treatment related behavioural skills and cognitive strategies in relevant real-life contexts^27^.

### Sample size

The sample size for this study was set to provide sufficient qualitative data to explore the feasibility, acceptability and fidelity of the STAMINA intervention^18^ and as such a formal power calculation was not appropriate. We planned to recruit up to 40 men on ADT from up to 4 NHS sites and sample 32 audio recordings.

### Data analysis

A combination of qualitative and quantitative measures were used to address the aim of the study.

#### Feasibility

Summary statistics were produced in Microsoft Excel, for eligibility, recruitment, retention, exercise programme adherence and safety, and are presented as mean ± SD. The magnitude difference between exercise test duration at baseline and 12 weeks was calculated using Cohen’s D and presented as an effect size.

#### Acceptability

Audio recordings were transcribed verbatim, checked for accuracy, and analysed deductively in Microsoft Excel, following the five stages of framework analysis^28^ (ES). The analysis began by reading the interview transcripts then considering the relevance of the text to the domains of the Theoretical Framework of Acceptability (TFA). Text was attributed to more than one domain where applicable and data was coded inclusively to maintain context throughout the analysis (i.e. text before and after the quote of interest was coded)^29^. In the final stages of analysis, each domain was described, and findings interpreted. Twenty five percent of coding was cross checked by a second and third independent reviewer to reduce researcher bias (RT, SR). Consensus on coding and interpretation was achieved via discussion.

#### Fidelity

A purposive sample of 32 audio recordings, reflecting different professionals (i.e. HCPs and ETs), different sessions (i.e. first, middle or last session) and different sites were rated by two independent researchers using checklists that were in development in line with guidance^27^ (RT, SR, LS). Checklists included delivery and quality of delivery of target behaviours and BCTs specific to the HCP and ET role (See supplementary file 4). Delivery of each target behaviour and BCT were scored a maximum of 2 points (0 = poor, 1 = limited, 2 = good). Scores were combined and converted to a percentage to calculate an overall fidelity score. Levels of fidelity were reported in line with the literature as: 80–100% adherence interpreted as 'high fidelity', 51–79% as ‘moderate’ and 0–50% as ‘low’ fidelity^30,31^. BCTs were advocated for use when patients expressed ambivalence or resistance towards exercise, and thus were not always applicable/ included in the analysis. Consensus on inclusion of BCTs was reached through discussion with LS, who has extensive experience in the coding of BCTs.

## Results

### Feasibility

#### Participant recruitment and retention

We recruited three NHS sites (located in South Yorkshire, Derbyshire and North Somerset) and three community exercise partners between May and October 2019. Within this timeframe, a total of 33 HCPs were approached to take part in the STAMINA intervention due to their role in the care of men with prostate cancer on ADT. Twenty-six (79%) provided written informed consent to take part in the intervention and remained within their role until study closure. Four (12%) did not engage with emails from the research team and/ or the principle investigator at site and three (9%) had insufficient time to participate. In total, 26 HCPs were recruited and provided written consent to deliver the STAMINA intervention, including: Consultant Urologists (n = 8), Clinical Oncologists (n = 3), Clinical Nurse Specialists (n = 10), an Urological radiographer (n = 1), research nurses (n = 3) and a support worker (n = 1).

In parallel, a total of 24 ETs were approached to deliver the STAMINA intervention. Twenty (83%) provided informed consent before attending training, and four (17%) declined, due to insufficient time to participate. Four ETs later withdrew from the study due to personal reasons (n = 2) or termination of employment at Nuffield Health (n = 2). In total, 20 ETs were recruited to deliver the STAMINA intervention, including: community gym site managers (n = 2), fitness managers (n = 3), a physiologist (n = 1) and personal trainers (n = 14).

Between June 2019 and January 2020, forty-five men were eligible following initial screening and were provided with STAMINA study details. Three (7%) men declined participation and two (4%) men were subsequently excluded due to ineligibility. Of the remaining 40 men who completed further screening and baseline assessments, 36 (90%) were eligible to receive the STAMINA intervention. Following referral, one man (3%) withdrew due to difficulties managing the side effects of radiotherapy (and did not attend any exercise sessions) leaving 35 men (97%) on ADT for prostate cancer who consented to receive the STAMINA intervention (Table 1).

**Table 1:** Baseline characteristics of men on ADT in the STAMINA trial.

Baseline measures	
Age (mean ± SD)	72 ± 9 years
BMI (mean ± SD)	28.6 ± 4.2 kg m^2^
Resting heart rate (mean ± SD)	69 ± 12 beats per minute
Diastolic BP (mean ± SD)	77.4 ± 10.1 mmHg
Systolic BP (mean ± SD)	137.4 ± 13.4 mmHg
ADT duration (mean ± SD)	7 ± 9 months
Ethnicity	100% self-report as White British or English
Chair sit-to-stand test (mean ± SD)	12 ± 4 reps
Self-report health status	Poor health (n = 0; 0%) Unhealthy (n = 1; 3%) Moderately healthy (n = 9; 25%) Relatively good health (n = 26; 72%)
Self-report functional capacity	Get in and out of an armchair (n = 36; 100%) Leave the house independently (n = 36; 100%) Climb three flights of stairs unaided (n = 33; 92%) Walk 100 yds (91 m) without stopping (n = 34; 94%) Walk 1 mile (1.6 kms) without stopping (n = 28; 78%) Jog 100 yds (91 m) without stopping (n = 21; 58%) Jog 1 mile (1.6 kms) without stopping (n = 5; 14%)
Registered disabled	Yes, n = 1 (3%)
Smoker	Yes, n = 3 (8%)
Drink alcohol	Never (n = 3; 8%) Occasionally (n = 27; 75%) Once a day (n = 5; 14%) More than once a day (n = 1; 3%)

**Figure 2:**
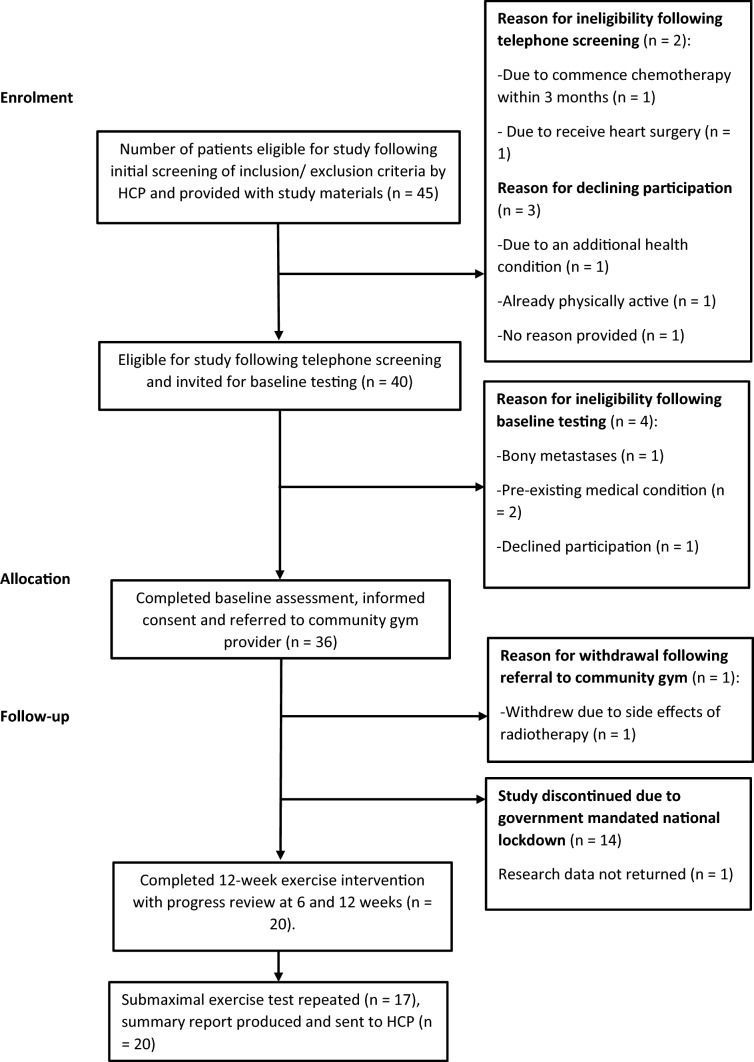
STAMINA trial CONSORT diagram: Patient recruitment and retention.

The study was discontinued early due to the coronavirus pandemic. Furthermore, one participant’s exercise data was not returned. Data presented here are on the men who completed the 12-week intervention and returned data (n = 20) before a government mandated national lockdown was enforced in the UK from March 2020 (Fig. 2).

#### Study adherence

**Figure 3:**
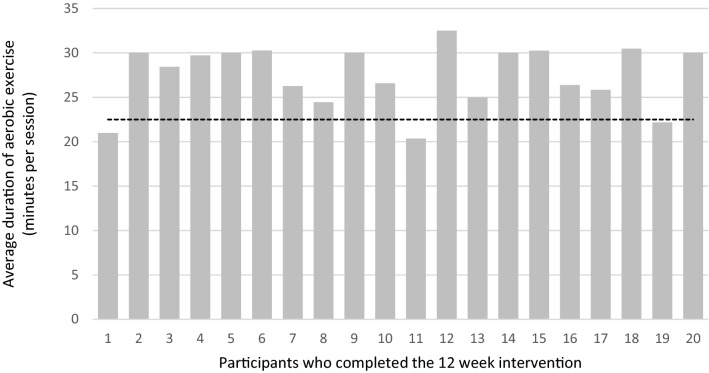
Average duration of aerobic exercise per session, in minutes for 20 participants. (Dashed line = 75% adherence or 22.5 min per session.)

Twenty men on ADT completed the STAMINA intervention, defined as completion of baseline and 12-week assessment in the NHS and 12 weeks of supervised exercise at the community-gym provider. Participants attended an average of 20 out of 24 scheduled supervised exercise sessions over 12 weeks (i.e. 83% of target). Reasons for non-attendance included: hospital appointments (4%), feeling unwell (18%), holidays (33%) and scheduling difficulties (46%). 25% of men were allocated a run-in period lasting between 4 and 6 weeks. Modifications were made to the frequency of sessions, from twice to once a week (n = 4), and to the type of exercise delivered, with one man starting with aerobic exercise only. In each prescribed session participants completed an average of 27.48 min (SD = 3.48) of aerobic exercise at 77% heart rate max (or RPE of 5), equating to 92% of the prescribed dose (Fig. 3). Furthermore, participants completed 3 sets (SD = 0.41) of 10 reps (SD = 0.65) of 3 (SD = 0.90) resistance exercises per session.

**Figure 4:**
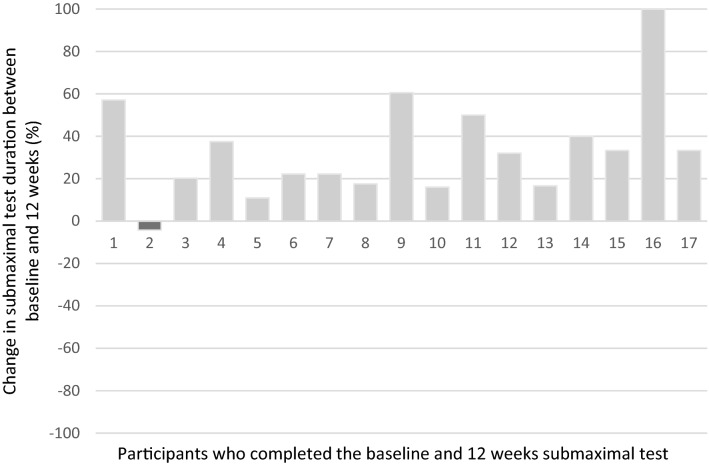
Percentage change in submaximal test duration between baseline and 12 weeks for 17 participants.

Seventeen (85%) men completed the exercise test at baseline and 12-weeks. Three participants were unable to repeat the test at 12 weeks due to feeling unwell: one participant reported fatigue from radiotherapy treatment and two were unrelated to treatment or the disease. At baseline, participants achieved a mean of 6.56 min (SD = 2.71) in the exercise test. A 33% (*d* = 0.66) increase in test duration was demonstrated at 12 weeks (mean = 8.40 min; SD = 2.85), with minimal change in final heart rate (baseline = 133.00 ± 24.36; 12 weeks = 134.18 ± 18.57) and RPE (baseline = 5.94 ± 1.71; 12 weeks = 6.35 ± 1.66) (Fig. 4).

Feedback to clinical teams was high with ETs completing a summary report for all (100%) participants. Only 5 out of 20 summary reports were discussed in follow-up care (i.e. 25% of target).

#### Adverse events

One serious adverse event, unrelated to the study, was reported during the intervention. The participant fainted at home, fracturing his ankle. Subsequent diagnostic tests were performed (i.e. echocardiogram and 24 h ECG) and reviewed by a cardiologist. The participant was deemed safe to continue exercising, and thus on healing of the injury, resumed the intervention. Additionally, four adverse events from three participants were reported over the duration of the trial. One was related to participation in the study and involved mild soft tissue pain of the left knee during a step-up exercise which recovered with rest between sessions. The other three adverse events were unrelated to study participation (i.e. bruising from a fall at home, pain from a catheter and pain from gout).

### Acceptability

Acceptability was explored in 22 planned interviews with participants who delivered (HCPs, n = 8; ETs, n = 6) and received the intervention (patients n = 8). Twelve interviews were conducted face-to-face and 10 via telephone. Selected quotes are presented in the 7 themes of the TFA below^26^ (see Supplementary 5 for full details).

#### Affective attitude

The men provided overwhelmingly positive reports of their experiences of participating in the STAMINA intervention, regardless of previous exercise experience. Positive reports were echoed by HCPs and ETs:

Yeah it was great actually because it was, for once it was something positive that we could offer to patients (HCPW1)

I just think it’s been phenomenal for their overall wellbeing (ETW1)

Excellent, excellent, from start to finish (PtC4)

#### Burden

The men thought that twice weekly supervised sessions were achievable, and some were keen to complete additional independent exercise sessions. HCP and ETs required time to attend professional training but mostly felt they had sufficient time to provide behavioural support, embedded into their current role:

When we initially see the patients to start them on the hormone treatment, I’m talking through the possible side effects with them, so I’m already mentioning to them, you know, and to counteract these they need to be fit and active (HCPC1)

Been really easy actually to get them all booked in (ETC3)

I was looking forward to it. Never one day did I feel it was a bind (PtS5)

#### Ethicality

The intervention aligned with all participants values including their professional development goals and personal beliefs related to the importance and beneficial effects of exercise for men on ADT:

I think we all really believe in it, because it’s an area that has been lacking for patients in terms of information and advice, but also resource (HCPS1)

We are here to help people. So yeah, I think it’s definitely the right kind of gym to do it. And the right range of trainers as well (ETC3)

When I was diagnosed originally, no I’d never give exercise a thought. If you’d said to me six month ago you’d be going to the gym I’d have laughed at you … but as Dr [name] told me, it’s manageable (PtC4)

#### Intervention coherence

All participants demonstrated a good understanding of the intervention components and intended study aims/ outcomes:

I think it gives them something positive. I think it allows them to take control (HCPS1)

So, we’ve seen cardiovascular improvements, strength improvements. I’m getting people coming to me going I’ve got a bit more energy now (ETC/S2)

If you go along with the right attitude and you’re prepared to put in as much effort as you people in getting it up together, you’ll get a lot out of it. (PtS5)

#### Opportunity costs

There were no perceived opportunity costs for patients. However, some clinic appointments were rescheduled to provide time for professional training and exercise delivery and some ETs extended their working hours to deliver the intervention off-shift in line with their preferences.

So, if we’re introducing a radiotherapy trial or a drug trial then we will spend more time in clinic talking to them about it. So, in a way it was no different (HCPW1)

For myself, it has meant some longer days (ETW1)

If I can’t find an hour and a half of my day to come and do this which is extending my life, that’s the way I look at it, I’d be a fool. I really would (PtS1)

#### Perceived effectiveness

All participants believe that supervised exercise has physical, psychological and social benefits for men on ADT, including a reduction in fatigue and improvements in QoL.

It was just something positive that we could say look we’ve got evidence that actually if you do supervised exercise then that may reduce the fatigue and improve your quality of life (HCPW1)

Psychologically it gets them out the house doesn’t it, so they’re not isolated. It gets them into an environment with a lot of people (ETC1)

It’s made a complete difference to my life in general, my head you know, everything. I look forward to everything (PtC4)

#### Self-efficacy

Some participants expressed initial apprehensions about delivering or participating in the intervention, however these were quickly addressed via professional training, experience, and ongoing support:

Not very. More confident now yeah, because I’ve got used to doing it (HCPC1)

With my particular role I felt OK, because that’s something that I do every day (ETS1)

I thought I wouldn’t be able to get anything out of it because of my [co-morbidity] but I was wrong. (PtC8)

### Fidelity

A total of 78 interactions between patients and HCPs (n = 31), and patients and ETs (n = 47), were digitally audio-recorded. A purposive sample of 32 audio recordings were analysed reflecting interactions at referral (n = 16), induction to the gym (n = 6), six (n = 6) and twelve-week review (n = 4). HCPs at one NHS trust did not audio record any of their interactions with patients in live time. As such, these specific audio recordings could not be coded for fidelity or included in the analysis. Furthermore, all of the interactions between patients and ETs were marked down during the analysis due to a lack of clarity on the audio recording of whether the behaviour had been performed and/ or of its quality as it was delivered partly or wholly outside of the clinic room (i.e., the treadmill/ bike exercise test).

Fidelity of the HCP and ET role were found to be moderate (74%) and high (82%), respectively^26^. Professionals scored more highly for the delivery, and quality, of target behaviours compared to the delivery, and quality, of applicable BCTs (Table 2).

**Table 2:** Fidelity of HCP and ET audio recorded consultations with men on ADT.

	HCP (n = 16)	ET (n = 16)	Total (n = 32)
Delivery of target behaviours^a^	77%	88%	81%
Quality of target behaviour delivery^a^	76%	84%	80%
Delivery of behaviour change techniques^a^	62%	77%	73%
Quality of behaviour change technique delivery^a^	44%	70%	65%
Total level of adherence^b^	74% (moderate)	82% (high)	78% (moderate)

^a^Delivery, and quality of delivery of target behaviours and behaviour change techniques were scored a maximum of 2 points each (0 = poor delivery; 1 = limited delivery; 2 = good delivery). Scores were combined and converted to a percentage (high fidelity = 80–100%; moderate fidelity = 51–79%; low fidelity = 0–50%).

^b^Scores for delivery of target behaviours, quality of target behaviour delivery, delivery of behaviour change techniques and quality of behaviour change technique delivery were combined and converted to a percentage to provide an overall fidelity score (high fidelity = 80–100%; moderate fidelity = 51–79%; low fidelity = 0–50%).

## Discussion

STAMINA is the first study to report on the feasibility and acceptability of a supervised exercise programme embedded into standard NHS prostate cancer care. We demonstrate that National Institute of Health and Care Excellence recommendations (NG131 1.4.19) can be delivered, by embedding exercise into the prostate cancer care pathway, in collaboration with community partners, thus addressing the large disconnect between what guidelines recommend as optimal care, and what patients receive. Furthermore, we demonstrate good feasibility, acceptability and fidelity in terms of recruitment, retention, adherence and delivery of behaviourally informed exercise support, with very few adverse events.

Our intervention design has provided preliminary data to suggest good uptake and retention to a supervised exercise programme embedded into prostate cancer care. Similar, findings have been demonstrated when supervised exercise is delivered in the community to breast cancer survivors^32^. However, other community-based exercise programmes, such as cardiac rehabilitation, have experienced poor uptake^33^, retention^34^ and adherence^33^ with only half of patients being recommended exercise by their clinical team. The STAMINA intervention sought to address limitations observed in cardiac rehabilitation by providing skill-based professional training to recommend and refer men on ADT for exercise. For example, we identified and addressed HCP barriers^6^ related to endorsing exercise and providing behavioural support to men on ADT. Subsequently, HCPs believed the intervention was likely to be effective and high levels of recruitment and retention were reported. For instance, all eligible participants completed the full intervention where possible, with the exception of one participant who discontinued due to side effects of radiotherapy, and this was very early on, i.e., after one session.

The exercise prescription was considered acceptable in a community-gym environment by men on ADT, with twice weekly supervision deemed the optimal frequency of exercise training. Adherence to the exercise prescription was high, with most men achieving the prescribed exercise dose (intensity and volume) and attending most scheduled sessions. Some participants were allocated a tailored run-in period, demonstrating community-based ETs skills to deliver a bespoke, safe and effective exercise programme based on an individual’s capability, opportunity and motivation^24^. Similar results were previously reported in two RCTs delivering combined aerobic and resistance training with^8^ or without^11^ dietary advice to men on ADT for prostate cancer. The high levels of adherence to, and acceptability of, the present intervention suggest no alterations to the exercise prescription are required ahead of the planned definitive RCT. Furthermore, results from the submaximal exercise test imply that the exercise prescription had a positive impact on physical fitness and endurance which may reduce the decline in physical function observed with ADT^35^ in line with what has previously been reported in systematic reviews^15^.

Overall, the intervention content was delivered with moderate to high fidelity, i.e., HCPs and ETs demonstrated good application and delivery of the skills learnt in training into real-life context. This in turn may facilitate the implementation of our behaviour change intervention into clinical practice^36^ and provide confidence that changes in study outcomes will likely be due to the intervention and not variability in implementation^37^. However, as demonstrated previously^38^ we identified an inherent limitation in the data capture technique and subsequent analysis. For example, acceptability of the measurement approach (particularly in HCPs) was poor, with audio recordings acting as a barrier to intervention delivery, indicating a clear division between research and practise, as previously reported^39^. Therefore, fidelity of the intervention and acceptability of the research tool may be improved by designing an additional/ alternative measure that considers both psychometric (i.e. validity and reliability) and implementation qualities (i.e. acceptability and user-friendliness)^40^, or assessing fidelity post training using role model scenarios.

Embedding exercise into the prostate cancer care pathway was deemed highly acceptable by participants who delivered and received the intervention, aligning with their values and beliefs about exercise as a treatment component^26^. Through experience and professional training, HCPs and ETs were able to integrate behavioural support into routine clinic discussions and exercise sessions confidently, thus demonstrating the importance of professional behaviour change facilitated by intervention development approaches^24^. Our acceptability data lends considerations for implementation. For example, managerial support and time is required to attend professional training, and ETs require protected time to deliver the intervention on shift to reduce extensive working hours. The next step is to evaluate the clinical and cost effectiveness of embedding exercise into routine prostate cancer care in a planned definitive trial exploring maintenance of exercise and long-term behaviour change.

There are limitations to the present study that should be noted. This was a non-confirmatory, non-randomised prospective cohort study and as such all data is to be taken as preliminary. Our findings are primarily for the purpose of intervention development, refinement, and optimisation. The study was discontinued early due to the coronavirus pandemic and thus 14 men were unable to complete the intervention. Furthermore, only a quarter of progress reports were discussed in NHS follow-up appointments due to variability of care (e.g., follow-up care delivered by a non-STAMINA trained HCP) and problems in the electronic communication pathway (e.g., difficulty uploading the progress report into patient notes). Also, we did not encourage men to generalise behaviours learnt in supervised exercise environments to other non-supervised contexts, which may influence long-term adherence and habit formation in a longer trial. Despite the limitations, we captured sufficient qualitative data to explore the feasibility, acceptability and fidelity of the STAMINA intervention as originally planned. However, the impact of societal changes due to the pandemic creates uncertainty regarding the future of face-to-face interventions. Telemedicine is anticipated to play an increasing role in healthcare and is rapidly being adopted by healthcare services with potential for extension to clinical exercise services for people with cancer^41^. Furthermore, delivery of remote supervised exercise could circumvent financial pressures of exercise services and provide a scalable service independent of residential location. However, currently there is insufficient evidence to support efficacy of remote exercise programmes and thus this warrants further exploration^42^.

## Conclusion

The STAMINA intervention, which is designed to embed clinical guidance around exercise training into standard prostate cancer care to optimise management of men on ADT demonstrated good feasibility and acceptability. Men on ADT reported positive impacts on lifestyle behaviours and measures of physical fitness and function. Furthermore, the intervention was deemed highly acceptable by HCPs, ETs and men on ADT. Some research processes, particularly around fidelity assessment, need to be refined. The intervention (with few adaptations) has been shown to be suitable for evaluation of clinical and cost-effectiveness in a planned definitive randomised clinical trial.

## Supplementary Information

Supplementary Information.

## Data Availability

All data generated or analysed during this study are included in this published article (and its Supplementary Information files).
