# Supplementary material for: Embedding supervised exercise training for men on androgen deprivation therapy into standard prostate cancer care: a feasibility and acceptability study (the STAMINA trial)
Source: Sci Rep. 2021 Jun 14;11:12470. doi: 10.1038/s41598-021-91876-y (PMC8203669; doi:10.1038/s41598-021-91876-y)
Supplement: Supplementary file 1 — Supplementary Information. [file 41598_2021_91876_MOESM1_ESM.docx]

**Title:** Embedding supervised exercise training for men on androgen deprivation therapy into standard prostate cancer care: A feasibility and acceptability study (The STAMINA trial)

**Authors:** Sophie Reale^1^, Rebecca R. Turner^1^, Eileen Sutton^2^, Liz Steed^3^, Stephanie J. C.Taylor^3^, Dylan Morrissey^4,^, Patrick Doherty^5^, Diana M. Greenfield^6^, Michelle Collinson^7^, Jenny Hewison^8^, Janet Brown^9^, Saïd Ibeggazene^1^, Malcolm Mason^10^ , Derek J. Rosario^11^ , Liam Bourke*^1^ and on behalf of the STAMINA co-investigators.

**Corresponding author:** Liam Bourke^1^, L.Bourke@shu.ac.uk

**Affiliations:**

^1^Allied Health Professionals, Radiotherapy and Oncology, Sheffield Hallam University, UK

^2^ Population Health Sciences, University of Bristol, UK

^3^ Institute for Population Health Sciences, Queen Mary University of London, UK

^4^ Sports and Exercise Medicine, William Harvey Research Institute, School of Medicine and Dentistry, Queen Mary University of London, London, UK

^5^Department of Health Sciences, University of York, UK

^6^Specialised Cancer Services, Sheffield Teaching Hospital NHS Foundation Trust, UK

^7^ Clinical Trials Research Unit, Leeds Institute of Clinical Trials Research, University of Leeds, UK

^8^ Leeds Institute of Health Sciences, School of Medicine, University of Leeds, UK

^9^ Department of Oncology and Metabolism, University of Sheffield, UK

^10^ School of Medicine, Cardiff University, UK

^11^Department of Urology, Sheffield Teaching Hospitals, UK

**Supplementary files**

**Supplementary file 1: Summary of HCP and EP training**

Template for Intervention Description and Replication of the healthcare professional intervention

| **Name** | Healthcare professional training package: Integrating exercise recommendations into prostate cancer clinical care |
| --- | --- |
| **Why?** | In 2014 the UK National Institute for Health and Care Excellence (NICE) incorporated a recommendation in the prostate cancer management guidelines that all men starting Androgen Deprivation therapy (ADT) should be offered 12 weeks of twice-weekly supervised aerobic and resistance exercise to reduce fatigue and improve quality of life. These recommendations do not reflect usual care. HCPs report several barriers to delivery of these recommendations and providing exercise support to men with prostate cancer on ADT. Therefore, a training package was developed to support HCPs in recommending exercise, providing behavioural support and exercise in line with recent NICE recommendations NG131 1.4.19. |
| **What?** | A half day, skill-based, interactive training package was developed, consisting of six modules.  **Module one: An overview of the project**  This module introduced the training facilitators to the clinical team, gave an overview of the training package and of the project. NICE NG131 1.4.19 recommendations were introduced here.  **Module two: Prostate cancer and exercise – the evidence base**  This module gave an overview of the benefits of exercise for men with prostate cancer on ADT.  **Module three: The role of the clinical team**  This module identified clinical roles within the team to aid the implementation of the NICE recommendations. Discussions and demonstrations around how to broach the topic of exercise and lifestyle with this patient group were included. Common assumptions made by HCPs about patients’ capabilities to exercise were addressed via the use of patient case studies and group tasks.  **Module four: Skills to supporting people with exercise**  This module provided HCPs with the appropriate skills in terms of behaviour change techniques to use to support this patient group with exercise. Role-play and group tasks were included within this module.  **Module five: The exercise referral pathway and communication pathway**  This module provided HCPs with the information of how to make referrals for exercise, what information to hand out to patients and how secure communication will take place with Nuffield Health and the NHS.  **Module six: Follow-up (Repeated at 8-12 weeks)**  This module provided HCPs with the appropriate skills to provide exercise support at follow-up. Role-play and group tasks were included within this module. |
| **Who provided?** | Behavioural science researcher/Health Psychologist.  Minimum of two facilitators. |
| **How?** | Face to face in small groups of up to 10. |
| **Where?** | On site at the hospital, a university building, or an off-site location such as a conference centre. |
| **When and how much?** | Half day training with module 6 repeated at 8-12 weeks. |
| **Tailoring?** | Care within prostate cancer is complex. Therefore, modules were assigned to HCPs depending upon their role within the prostate cancer care pathway e.g. Consultant Urologists who prescribe men ADT were required to attend module 1-3 whereas key workers who see men on ADT at diagnosis and follow up were required to attend module 1-6. |
| **Modifications?** | Face to face, delivered on NHS site or locally to NHS site. |
| **How well?** | Process measures, acceptability and fidelity were measured as part of a feasibility study. |

Template for Intervention Description and Replication of the exercise professional intervention

| **Name** | Exercise professional training package: Supporting men on ADT to exercise |
| --- | --- |
| **Why?** | The National Institute for Health Excellence (NICE) recommend that men on ADT should be offered twice weekly aerobic and resistance exercise to improve cancer specific fatigue and quality of life. However, these guidelines are not being met. Community-based exercise professionals are well suited to deliver these recommendations however due to their limited training and experience of working with clinical populations, further training is required. |
| **What?** | Exercise professionals received two online modules and one full day skill-based, interactive training package consisting of six modules.  Exercise professionals were provided with a training manual, PowerPoint slides (online content and paper format), video examples of all behaviours, prompt sheets, case studies and associated worksheets to facilitate activities centred on tailoring exercise and writing progress reports.  **Level 1 training**  Module 1: Introduction to prostate cancer and exercise  This module provides introductory content on the symptoms, prevalence, treatments and side effects of prostate cancer and the evidence-base of exercise as a treatment component.  Module 2: Operationalising the STAMINA programme  This module provides content on scheduling patient appointments and the referral/ communication pathway between the exercise professionals and clinical team.  **Level 2 training**  Module 1: Working with clinical populations  This module focusses on developing an understanding of the target population (i.e. men with PCa on ADT). We discuss side effects, beliefs towards exercise, feelings and emotional state, previous experiences in a gym environment and demonstrate active listening skills.  Module 2: Tailoring the exercise prescription  This module covers the skills and knowledge required to tailor exercise prescriptions for clinical populations. Evidence based information is provided as well as skill-based learning with feedback.  Module 3: Delivering the exercise prescription  This module is centred on delivering tailored exercise programmes one-to-one and in small groups. We discuss the importance of monitoring exercise and practise the required skills using graded tasks and feedback.  Module 4: Reviewing the exercise prescription  This module prepares exercise professionals to review patient progress weekly and more formally at 6 and 12 weeks. Example videos are provided and exercise professionals practise completing progress reports in a role play task. Furthermore, we explore good communication skills.  Module 5: Behaviour change  This module focussed on behaviour change theory and behaviour change techniques to support initiation and maintenance of exercise, as well as ambivalence and resistance to exercise. Role play tasks are intertwined with case study examples and group discussion.  Module 6: Fitness testing  This module prepares exercise professionals to conduct a submaximal fitness test. Instruction and demonstration are provided before providing exercise professionals with time to practise and receive feedback. Exercise professionals also practice completing the required paperwork for recording results of the fitness test and practise explaining the purpose of the test in a role play task. |
| **Who provided?** | - Lead facilitator - Behavioural Science Research Fellow with a background in Sport and Exercise Science, Psychology and Public Health. - Co-facilitator - lead personal trainer from Nuffield Health with a background in biomechanics and many years of experience tailoring and supervising exercise for clinical populations. |
| **How?** | The training package was delivered individually, online (Level 1) and face-to-face (Level 2) in small groups ranging from 2 -10 people. |
| **Where?** | On site at community exercise gyms with access to an education room (tables, chairs, projector and screen) and equipment on the gym floor for practical sessions. |
| **When and how much?** | Exercise professionals completed level 1 and 2 once. Level 1 was administered four weeks before the scheduled level 2 training to provide sufficient time for completion. Non-completion or < 80% pass mark on the multiple-choice test in level 1, prevented exercise professionals advancing to level 2 training. |
| **Tailoring?** | No tailoring was required. |
| **How well?** | Process measures, acceptability and fidelity were measured as part of a feasibility study. |

**Supplementary file 2: Participant inclusion and exclusion criteria**

Health care professionals

Inclusion criteria for HCPs:

- Involved in diagnosis and or treatment and or follow-up of men with prostate cancer on ADT
- Able and willing to receive draft intervention training for HCPs as developed in STAMINA WP2
- Based at a site with sufficient number of men started on long-term ADT to achieve recruitment target within timelines.

Exclusion criteria for HCPs:

- No active involvement in the management of men with prostate cancer on long term ADT
- Not based at a site with sufficient number of men started on long-term ADT to achieve recruitment target within timelines

Exercise professionals

Inclusion criteria for EPs

- Degree in sport and or exercise science
- Registered as a level 3 PT
- Experience in working with clinical populations
- 1 year continuous service with employer and meeting employers performance targets
- Experience of delivering fitness testing protocols
- Pass mandatory STAMINA assessment by at least 80%

Exclusion criteria for EPs:

- Insufficient evidence of working with clinical populations previously
- No relevant qualifications or professional registration
- Not meeting employers performance targets in their role
- Failing STAMINA mandatory assessment

Men on ADT

Inclusion criteria for men on ADT:

- Men with prostate cancer on ADT
- Willing and able to provide informed consent
- Men not due to receive chemotherapy within 3 months or those having completed chemotherapy

Exclusion criteria for men on ADT:

- Men with metastatic castrate resistant prostate cancer
- Men due to commence chemotherapy within 3 months
- Unable to provide informed consent (lack capacity)
- Those not able to complete study assessments
- Men with unstable angina
- Uncontrolled hypertension and/ or diabetes mellitus
- Recent myocardial infarction (within past 6 months)
- Painful or unstable bony metastases
- Fixed output pacemakers
- Any other absolute contraindication to exercise as defined by clinical guidance e.g. ACPICR standards.
- Those with any pre-existing medical condition that would prevent safe tailoring of exercise which is not likely to be resolved in the work package 3 time frame

# Supplementary file 3: Topic guides to explore acceptability of the HCP, EP and patient intervention

# 3A. Topic guide for interviews with men who have received the exercise intervention in work package 3 (Based on Sekhon 2017)

- How did you find the STAMINA programme ?
  - Experience of the intervention?
  - Feelings about the intervention?
    - What did you like/didn’t you like about the programme?
- What did participation in the intervention involve for you?
  - What did it cost you in terms of time/ opportunity/ effort to participate in the intervention?
  - Did you have to give anything up in order to participate in the intervention?
  - If you discontinued the intervention, why do you do this?
- How does the intervention fit in, or match up with, what you feel would be helpful to you?
  - Do you believe this sort of intervention can be helpful to men in your situation?
- What do you think about the actual content of the intervention?
  - i)The conversation/ advice / encouragement from the doctors and nurses looking after you at the hospital before you started the intervention
  - ii)The gym components
  - iii) The follow up by the doctors and nurses looking after you at the hospital after you had started the gym visits
  - Could the intervention be improved? If so, how?
- How confident were you about doing the intervention?
  - At the start?
  - Now
  - Do you have any concerns or worries about the intervention?
    - Probe the three components (i-iii) mentioned above
- How do you think men like yourself you might benefit from the intervention?
  - Probe the three components (i-iii) mentioned above
- Do you feel the intervention was helpful to you?
  - How did you benefit from it?
    - Fitness?
    - Other health benefits?
    - Social benefits?
  - If you didn’t find it helpful, why not?
  - Do you think you could stick to the exercise programme if it were offered over a longer period? (e.g. to 12 months and beyond)?
  - Would there be any barriers to you attending a programme for a longer period?
  - What kind of things would help you, or put you off attending a longer programme?
- Is there anything else you’d like to say about your experience of the intervention or about the intervention in general?

# 3B. Topic guide for interviews with personal trainers and exercise physiologists who have delivered the exercise intervention in work package 3 (Based on Sekhon 2017)

- How did you find delivering the STAMINA programme?
  - Experience of delivering the STAMINA programme?
  - What format, one-to-one or group drop in sessions?
  - Feelings about the STAMINA programme?
- What did delivering the STAMINA programme involve?
- What did it involve in terms of time/ opportunity/ effort to train to, and then deliver, the STAMINA programme?
  - Measuring heart rate and rate of perceived exertion/ recording data at 5 minute intervals/ tailoring the exercise prescription/ accessing nhs.net email/ audio recording consultations
  - What did you think of the training you received (content, format, length)
- Do you believe this sort of programme can be helpful to men with prostate cancer?
  - In what way?
- If you believe the STAMINA programme is beneficial, how do you think it might work?
- Do you feel the role of delivering the STAMINA programme is appropriate for exercise professionals such as yourself?
  - Do you have any concerns about this role?
  - Who should deliver the STAMINA programme, PT's or physiologists?
- How does the STAMINA programme fit in with your professional development?
- What do you think about the actual content of the STAMINA programme?
  - i)The conversation/ advice / encouragement from the doctors and nurses before participant patients start
  - ii)The gym components
  - iii) The follow up by the doctors and nurses looking after you at the hospital after you had started the gym visit
  - Could the STAMINA programme be improved? If so, how?
- How confident were you about delivering the STAMINA programme?
  - At the start?
  - Now
  - Did you feel you had enough support to deliver?
  - Is there anything else that would be helpful?
- Do you have any concerns or worries about the STAMINA programme?
  - Probe the three components (i) to iii)) mentioned above
- Is there anything else you’d like to say about your experience of delivering the STAMINA programme or about the STAMINA programme in general?

# 3C. Topic guide for interviews with members of the multidisciplinary clinical team caring for men who have received the exercise intervention in work package 3 (Based on Sekhon 2017)

- How did you find delivering the STAMINA Programme?
  - Experience of delivering the STAMINA Programme
  - Feelings about the STAMINA programme?
- What did delivering the STAMINA programme involve?
  - What did it involve in terms of time/ opportunity/ effort to train to, and then deliver, the programme?
  - Referral processes/eligibility criteria/screening log/audio recording/progress reporting/communications with EPs
- Do you believe this sort of programme can be helpful to men in with prostate cancer?
  - In what way?
- If you believe the STAMINA programme is beneficial, how do you think it might work?
- Do you feel the role of delivering this programme is appropriate for members of the MDT?
  - Do you have any concerns about this role?
- How does delivering the exercise support components of the programme fit in with your professional development?
- What do you think about the actual content of the STAMINA programme?
  - a) The gym components
  - b) How follow-up is embedded in the STAMINA programme?
  - Could the STAMINA programme be improved? If so, how?
- How confident were you about delivering the STAMINA Programme?
  - At the start?
  - Now
- Do you have any concerns or worries about the STAMINA Programme?
  - Probe the two components (a - b) mentioned above
- Did you feel that you have enough support to deliver the programme?
  - Show the HCP the draft booklet and ask for comments
  - Probe whether the table is useful, should it sit in the manual or appendices?
- Is there anything else you’d like to say about your experience of delivering the STAMINA Programme or about the programme in general?

# References

Sekhon M, Cartwright M, Francis JJ. Acceptability of healthcare interventions: an overview of reviews and development of a theoretical framework. BMC Health Services Research, 2017, Volume 17, Number 1, Page 1 <https://doi.org/10.1186/s12913-017-2031-8>

**Supplementary file 4: HCP and EP Fidelity checklists**

**4A. Healthcare professionals’ initial consultation with men on ADT**

**General instructions**

There are three key aspects to this fidelity checklist:

1) Record fidelity of the necessary behaviours

2) Assess the quality of behaviours

3) Assess the quality of behaviour change techniques (if used)

| **Recording identifier** |  |
| --- | --- |

|  | **Scores** | **Overall fidelity % score** |
| --- | --- | --- |
| **Behaviours scoring** |  |  |
| **Quality of behaviours** |  |  |
| **BCT delivery** |  |  |
| **Quality of BCTs** |  |  |
| **Overall score** |  |  |

| **Behaviours** | **Adherence to the content**  **(0 = No, 1 = Partially, 2 = Yes)** | **Quality of content** | **Quality of content** |
| --- | --- | --- | --- |
| Provide information on the side-effects of ADT |  | **0**  **1**  **2** | 0 = Little information given  1 = Limited information given, some key aspects missed  2 = Good information provided |
| Recommend exercise training as treatment component |  | **0**  **1**  **2** | 0 = Poor, little discussion  1 = Limited discussion around the benefits of exercise  2 = Good discussion on the different benefits of exercise as a treatment component |
| Provide information about the research processes e.g. discussions about referral process |  | **0**  **1**  **2** | 0 = Little information given  1 = Limited information given, some key aspects missed  2 = Good information provided |
| Provide information about the exercise referral scheme |  | **0**  **1**  **2** | 0 = Little information given  1 = Limited information given, some key aspects missed  2 = Good information provided |
| Ask if there are any barriers to exercise training |  | **0**  **1**  **2** | 0 = Poor, with closed questions  1 = Limited questions to explore barriers  2 = Good open questions |
| ***If applicable*** discuss any barriers and or facilitators to exercise training |  | **0**  **1**  **2** | 0 = Poor, with a lack of focus on barriers and facilitators  1 = Limited discussion around barriers and facilitators, not patient led  2 = Good discussion around barriers and facilitators. Participant led approach. |
|  | **Total score =** | **Total score =** |  |

| **Behaviour change techniques** | **Applicable or not?**  **(Yes or No)** | **Adherence to the content**  **(0 = No, 1 = Partially, 2 = Yes)** | **Quality of the content** | **Quality key** |
| --- | --- | --- | --- | --- |
| 1.2 Problem solving |  | **0**  **1**  **2** | **0**  **1**  **2** | 0 =Poor, attempting to problem solve for the patient and or problems identified but no solutions made.  1 = Prompting the patient to problem solve, but limited support for the patient to come up with solutions.  2 = Good interaction, supporting the patient to identify barriers themselves and come up with solutions. Led by patient, reflected by healthcare professional |
| 9.2 Pros and cons |  | **0**  **1**  **2** | **0**  **1**  **2** | 0 =Poor, attempting come up with pros and cons for the patient.  1 = Prompting the patient to come up with pros and cons, but limited support for the patient to come to a decisional balance  2 = Good interaction, supporting the patient to identify pros and cons and come to a decisional balance. Led by patient, reflected by healthcare professional |
| Outlining necessities and concerns |  | **0**  **1**  **2** | **0**  **1**  **2** | 0 = Poor, concerns are highlighted with little discussion around the necessity beliefs.  1 = Limited necessity and concerns factors are discussed and is not patient led.  2 = Good discussion, both concerns and necessities are discussed. |
| 15.1 Verbal persuasion about capability |  | **0**  **1**  **2** | **0**  **1**  **2** | 0 = Poor, no persuasion is given  1 = Limited verbal persuasion is given  2 = Good verbal persuasion is given; focus is on the positive aspects the patient can achieve. |
| 3.1 Providing social support |  | **0**  **1**  **2** | **0**  **1**  **2** | 0 = Poor, little attention given to the support that could be provided or advised on.  1 = Social support examples are discussed briefly  2 = Social support examples are provided and discussed with the patient |
| 6.3 Information about others' approval |  | **0**  **1**  **2** | **0**  **1**  **2** | 0 = Poor, information given is fear-inducing  1 = Limited discussion around the clinical teams approval for the patient to exercise  2 = Good discussion, it is explained to the patient that the clinical team approve and reasons why. Patient understanding is checked. |
| 5.1 ,5.3, 5.6 Information about consequences (health, emotional, social and environmental) |  | **0**  **1**  **2** | **0**  **1**  **2** | 0 = Poor, information given is fear-inducing  1 = Limited information is provided  2 = Helpful information is provided, checking for patient understanding |
|  |  | **Total score =** | **Total score =** |  |

| **Any other comments** |
| --- |
|  |

**4B. Induction to community-based gym with exercise professional**

**General instructions**

There are three key aspects to this fidelity checklist:

1) Record fidelity of the necessary behaviours

2) Assess the quality of behaviours

3) Assess the quality of behaviour change techniques (if used)

| **Recording identifier** |  |
| --- | --- |
| **Fidelity scorer (initials)** |  |
| **Date of fidelity score** |  |

|  | **Scores** | **Overall fidelity % score** |
| --- | --- | --- |
| **Behaviours scoring** |  |  |
| **Quality of behaviours** |  |  |
| **BCT delivery** |  |  |
| **Quality of BCTs** |  |  |
| **Overall score** |  |  |

| **Behaviours** | **Adherence to the content**  (0 = Not complete, 1 = Partially complete/ unclear from audio recording, 2 = completed) | **Quality of content** | **Quality of content key** |
| --- | --- | --- | --- |
| Provide a tour of the gym and facilities |  |  | 0 = Minimal tour provided; most key aspects missed  1 = Limited tour provided; some key aspects missed  2 = A clear and detailed tour of the gym and facilities |
| Explain membership details and access |  |  | 0 = Minimal information provided; most key aspects missed  1 = Limited information provided; some key aspects missed  2 = Clear and detailed information provided |
| Explore patient attitude and expectations |  |  | 0 = Minimal exploration  1 = Some exploration but limited follow up questions  2 = Full exploration with follow-up questions for detail |
| Undertake the submaximal exercise test |  |  | 0 = No rationale provided or deviation from protocol  1 = Limited rationale provided and test only partially complete  2 = Full rationale provided and test complete to protocol |
| Explain and agree the tailored exercise programme |  |  | 0 = Minimal information provided; most key aspects missed  1 = Limited information provided; some key aspects missed  2 = Clear and detailed information provided |
| Discuss and arrange time/ format of weekly supervised exercise sessions |  |  | 0 = Minimal information on session format, does not consider patient preference  1 = Limited information provided, some consideration to patient preference  2 = Clear and detailed information provided, and patient centred discussion on preferred format/ time |
| **Total score =** | | **Total score =** | |

| **Behaviour change techniques** | **Applicable or not?**  **(Yes or No)** | **Adherence to the content**  **(0 = No, 1 = Partially, 2 = Yes)** | **Quality of the content** | **Quality key** |
| --- | --- | --- | --- | --- |
| Outlining necessities and concerns |  | **0**  **1**  **2** | **0**  **1**  **2** | 0 = Poor, concerns are highlighted with little discussion around the necessity beliefs.  1 = Limited necessity and concerns factors are discussed and is not patient led.  2 = Good discussion, both concerns and necessities are discussed. |
| Pros and cons |  | **0**  **1**  **2** | **0**  **1**  **2** | 0 =Poor, attempting come up with pros and cons for the patient.  1 = Prompting the patient to come up with pros and cons, but limited support for the patient to come to a decisional balance  2 = Good interaction, supporting the patient to identify pros and cons and come to a decisional balance. Led by patient, reflected by healthcare professional |
| Problem solving |  | **0**  **1**  **2** | **0**  **1**  **2** | 0 =Poor, attempting to problem solve for the patient and or problems identified but no solutions made.  1 = Prompting the patient to problem solve, but limited support for the patient to come up with solutions.  2 = Good interaction, supporting the patient to identify barriers themselves and come up with solutions. Led by patient, reflected by healthcare professional |
| Importance ruler |  | **0**  **1**  **2** | **0**  **1**  **2** | 0 = Poor, attempting to identify placement on ruler for the patient  1 = Prompting the patient to identify a placement on the ruler, but limited exploration of patient placement  2 = Good interaction, supporting the patient to identify and explore rationale for the score provided |
|  | **Total score =** | | **Total score =** | |

| **Any other comments** |
| --- |
|  |

**4C. Six-week review at community-based gym with exercise professional**

**General instructions**

There are three key aspects to this fidelity checklist:

1) Record fidelity of the necessary behaviours

2) Assess the quality of behaviours

3) Assess the quality of behaviour change techniques (if used)

| **Recording identifier** |  |
| --- | --- |
| **Fidelity scorer (initials)** |  |
| **Date of fidelity score** |  |

|  | **Scores** | **Overall fidelity % score** |
| --- | --- | --- |
| **Behaviours scoring** |  |  |
| **Quality of behaviours** |  |  |
| **BCT delivery** |  |  |
| **Quality of BCTs** |  |  |
| **Overall score** |  |  |

| **Behaviours** | **Adherence to the content**  (0 = Not complete, 1 = Partially complete/ unclear from audio recording, 2 = completed) | **Quality of content** | **Quality of content key** |
| --- | --- | --- | --- |
| Discuss patient progress (outcome focussed) |  |  | 0 = No specific feedback provided and no exploration of patient perception of progress  1 = Some exploration of patient perception of progress  2 = Clear and detailed discussion of patient progress exploring both professional and patient views |
| Review and (where applicable) tailor the exercise prescription |  |  | 0 = No exploration of patient perception of exercise programme  1 = Some exploration of patient perception of exercise programme, limited tailoring where applicable with minimal rationale for change provided to the patient  2 = Clear and detailed discussion of the exercise prescription exploring both professional and patient views, and tailoring accurately where applicable (with full rationale for change provided to the patient) |
| Provide behavioural support |  |  | 0 = Incorrect use or frequently missed opportunities to use behaviour change techniques  1 = Mostly correct delivery of behaviour change techniques although more suitable techniques available  2 = Good timing and delivery of behaviour change techniques |
| Discuss the group supervised sessions available |  |  | 0 = Schedules or tells patient to attend group session without exploring patient perception or providing a rationale  1 = Limited exploration of patient perception on group sessions and some rationale for group-based exercise  2 = Clear and detailed information of the group sessions available and rationale for attending, with exploration of patient perception of group-based exercise. |
| **Total score =** | | **Total score =** | |

| **Behaviour change techniques** | **Applicable or not?**  **(Yes or No)** | **Adherence to the content**  **(0 = No, 1 = Partially, 2 = Yes)** | **Quality of the content** | **Quality key** |
| --- | --- | --- | --- | --- |
| Provide feedback on behaviour |  | **0**  **1**  **2** | **0**  **1**  **2** | 0 = Feedback is discouraging or not patient centred  1 = Feedback is generic and not specific to the patient  2 = Feedback is clear and constructive and specific to the behaviour |
| Discuss social support |  | **0**  **1**  **2** | **0**  **1**  **2** | 0 = Poor, little attention given to the support that could be provided or advised on.  1 = Social support examples are discussed briefly  2 = Social support examples are provided and discussed with the patient |
| Prompt self-monitoring |  | **0**  **1**  **2** | **0**  **1**  **2** | 0 = The patient is told they have to log their exercise  1 = Some information about the importance of self-monitoring is provided, limited discussion  2 = Clear and detailed information about the benefits of self-monitoring and discussion with patient to identify the most suitable method for them. |
| Discuss rewards |  | **0**  **1**  **2** | **0**  **1**  **2** | 0 = The patient is told what rewards to use and when  1 = Some information about the benefits of rewards is provided, limited discussion  2 = Clear and detailed information about the benefits of rewards and discussion with patient to identify the most suitable option for them. |
| Set SMART goals |  | **0**  **1**  **2** | **0**  **1**  **2** | 0 = The patient is told what their goal is  1 = Some discussion about a suitable goal for the patient but it does not follow all of the SMART principles, i.e. specific, measurable, achievable, relevant or time specific  2 = Clear and detailed discussion identify a goal that is patient centred and in line with SMART principles |
| Discuss habit formation |  | **0**  **1**  **2** | **0**  **1**  **2** | 0 = Unclear information about how habits are formed or the link to behaviour change maintenance  1 = Some discussion about how habits are formed with some information about the link to behaviour change maintenance  2 = Clear and detailed discussion about habit formation and behaviour change maintenance, exploring what ‘triggers’ that patients exercise behaviour |
| Pros and cons |  | **0**  **1**  **2** | **0**  **1**  **2** | 0 =Poor, attempting come up with pros and cons for the patient.  1 = Prompting the patient to come up with pros and cons, but limited support for the patient to come to a decisional balance  2 = Good interaction, supporting the patient to identify pros and cons and come to a decisional balance. Led by patient, reflected by healthcare professional |
| Problem solving |  | **0**  **1**  **2** | **0**  **1**  **2** | 0 =Poor, attempting to problem solve for the patient and or problems identified but no solutions made.  1 = Prompting the patient to problem solve, but limited support for the patient to come up with solutions.  2 = Good interaction, supporting the patient to identify barriers themselves and come up with solutions. Led by patient, reflected by healthcare professional |
| Importance ruler |  | **0**  **1**  **2** | **0**  **1**  **2** | 0 = Poor, attempting to identify placement on ruler for the patient  1 = Prompting the patient to identify a placement on the ruler, but limited exploration of patient placement  2 = Good interaction, supporting the patient to identify and explore rationale for the score provided |
|  | **Total score =** | | **Total score =** | |

| **Any other comments** |
| --- |
|  |

**4D. Twelve-week review at community-based gym with exercise professional**

**General instructions**

There are three key aspects to this fidelity checklist:

1) Record fidelity of the necessary behaviours

2) Assess the quality of behaviours

3) Assess the quality of behaviour change techniques (if used)

| **Recording identifier** |  |
| --- | --- |
| **Fidelity scorer (initials)** |  |
| **Date of fidelity score** |  |

|  | **Scores** | **Overall fidelity % score** |
| --- | --- | --- |
| **Behaviours scoring** |  |  |
| **Quality of behaviours** |  |  |
| **BCT delivery** |  |  |
| **Quality of BCTs** |  |  |
| **Overall score** |  |  |

| **Behaviours** | **Adherence to the content**  (0 = Not complete, 1 = Partially complete/ unclear from audio recording, 2 = completed) | **Quality of content** | **Quality of content key** |
| --- | --- | --- | --- |
| Discuss patient progress (outcome focussed) |  |  | 0 = No specific feedback provided and no exploration of patient perception of progress  1 = Some exploration of patient perception of progress  2 = Clear and detailed discussion of patient progress exploring both professional and patient views |
| Undertake submaximal test |  |  | 0 = No rationale provided or deviation from protocol  1 = Limited rationale provided and test only partially complete  2 = Full rationale provided and test complete to protocol |
| Complete summary report |  |  | 0 = No input from patient or sections missed  1 = Limited information provided  2 = Clear and detailed information provided based on discussion and input from patient |
| Discuss exercise maintenance and next steps |  |  | 0 = Patient is told what their next steps should be, not patient centred, no discussion of exercise maintenance  1 = Limited exploration of patient plans for the future, some information about exercise maintenance  2 = Clear and detailed information about exercise maintenance and balanced discussion about possible future exercise plans and how these can be supported |
| **Total score =** | | **Total score =** | |

| **Behaviour change techniques** | **Applicable or not?**  **(Yes or No)** | **Adherence to the content**  **(0 = No, 1 = Partially, 2 = Yes)** | **Quality of the content** | **Quality key** |
| --- | --- | --- | --- | --- |
| Provide feedback on behaviour |  | **0**  **1**  **2** | **0**  **1**  **2** | 0 = Feedback is discouraging or not patient centred  1 = Feedback is generic and not specific to the patient  2 = Feedback is clear and constructive and specific to the behaviour |
| Discuss habit formation |  | **0**  **1**  **2** | **0**  **1**  **2** | 0 = Unclear information about how habits are formed or the link to behaviour change maintenance  1 = Some discussion about how habits are formed with some information about the link to behaviour change maintenance  2 = Clear and detailed discussion about habit formation and behaviour change maintenance, exploring what ‘triggers’ that patients exercise behaviour |
|  | **Total score =** | | **Total score =** | |

| **Any other comments** |
| --- |
|  |

**Supplementary file 5:** Interview quotes mapped onto the Theoretical Framework of Acceptability

| **TFA Construct** | **Summary** | **Quotes** |
| --- | --- | --- |
| 1. **Affective Attitude**   (How an individual feels about the intervention) | The men provided overwhelmingly positive reports of their experiences of participating in the STAMINA intervention, regardless of whether they had recent experience of exercise or had a relatively sedentary lifestyle. | **PtC4**: “*Excellent from start to finish”*  **PtS1**: “*It’s great. It’s been a big benefit to me”*  **PtS5**: “*I felt more alive”*  **PtC8**: “*It was brilliant … it took me out of myself”*  **PtC4**: “*Excellent, excellent, from start to finish”*  **PtS7**: “*I’m very pleased to come down … very good, no doubt about it. And I always think how lucky I’ve been to get on this research thing”*  **PtW2**: “*I couldn’t fault it really. As far as I was concerned it worked just fine”*  **PtC12**: “*I got on very well actually, yes I did. I got on very well … I definitely felt some benefits from doing it as well”* |
|  | HCPs were pleased to have a supervised exercise programme to refer men to as no services were previously available, and they felt it meant they were finally able to offer the patient something positive. HCPs also enjoyed having the opportunity to participate in further professional training. | **HCPC1**: “*I think it’s an excellent programme. Because there are exercise programmes out there, 12-week programmes that people can be referred to, but we find out on the day it doesn’t actually encompass cancer patients.”*  **HCPS2**: “*I think it fits in correctly with the way we are in the setting in clinic where we first see the patient once they’ve had the diagnosis. It isn’t an extra add-on. And I don’t think it’s inappropriate to talk at that stage because as I say we’ve already mentioned that I do sometimes talk about diet with some of these patients. So, exercise is part of it.”*  **HCPC3**: “*It’s a good idea. I can’t tell you whether it’s going to help people or not, because that’s the whole point of doing the follow-on trial isn’t it and we think we did the pilot study. But yeah, it’s like any other idea, wait and see what the results are.”*  **HCPW1**: “*Yeah it was great actually because it was, for once it was something positive that we could offer to patients. So often we’re telling them about the side effects of treatments or that the treatments haven’t, sometimes we do have good news to tell people, but we were actually able to say we’re going to start you on this treatment, but here is a supported supervised exercise therapy that we can offer you to try and help reduce some of the side effects of the treatment.”*  **HCPW3**: “*I think it was very useful in terms of the education side of it. So, us learning a bit more about why the physical aspect, regarding the hormones and the ADT is important, and just re-educating ourselves, I guess, having a different conversation about what exercise is important. I think a lot of us had been saying exercise is important but not really having any emphasis behind that. So, I think the initial learning behind which exercises and also having that prescribed exercise was definitely an element that we thought was really useful. And by the consultants being able to say right this is what you need to do, it was almost like a prescription of exercise.”* |
|  | EPs believes that the STAMINA programme offered men on ADT a positive and enjoyable introduction to exercise with many beneficial outcomes including physical fitness and emotional wellbeing. EPs believe the intervention should be more widely accessible. | **EPC1**: “*With the feedback we’ve given, they’ve been really positive and they understand. Because we’ve got the experience of delivering these programmes, for example, we’re presently doing joint pain and cystic fibrosis as well, we understand the feedback really well and positive.”*  **EPW1**: “*I just think it’s been phenomenal for their overall wellbeing.*”  **EPC/S2**: “*I’d say generally I think it’s really positive. I’ve only had a few that I’ve actually ended up taking through the whole 12-week cycle so far, I think 18 still enrolled. But the feedback that we’re getting is really positive. They’ve all found it something really enjoyable that it’s given them a bit more of a focus and they’ve all found benefits in terms of fitness, but also some emotional wellbeing so far as well, which is really encouraging to hear. So, it’s been really positive overall”*  **EPC/S2**: “*Oh yeah, hundred percent definitely so. I’ve seen it first-hand and it definitely is helpful. And I’m not saying it from a physical point of view, but also an emotional wellbeing point of view, so yeah very good; overall definitely a positive experience for those men as well.”*  **EPS3***: “It’s good. Anything that gets people moving, even if it only helps one person it’s got to be good hasn’t it?”*  **EPS3**: “*If a lot of them don’t want to, they’d be the same population for everything isn’t there, there’d be people that would be quite positive about it and be training and doing that anyway. But for the ones that aren’t that positive it’s quite good to bring them in and show what can be done….. Whereas it’s the sort of thing that men just don’t really talk about it, let’s get on with it. So it’s quite, it would be quite good to have it more available I guess, so more in the public eye.”*  **EPC3**: “*I think my understanding of how they were going to be from the training and stuff was that, and no disrespect to the guys that are on the STAMINA project, I thought it was going to have to be a lot more basic and easy for them than what it is. But they’re quite, whether it’s just my guys, I think a few of the others, I’m not sure, but they’re a lot stronger than I perceived or a lot more fit than they think they are a lot of the time.”* |
| 1. **Burden**   (The perceived amount of effort that is required to participate in the intervention) | The men were comfortable with the level of effort that was required for participation in the intervention. Most thought that twice weekly supervised sessions were achievable, and some were keen to complete additional independent exercise sessions. | **PtC4**: “*I can fit that round [hobby] , because they open here at half past six in a morning probably until ten at night … if I can’t find an hour to come down here and do that well it’s a poor job ain’t it.”*  **PtC8**: “*Three times a week would have been too much but twice was ideal”*  **PtS5**: “*I was looking forward to it. Never one day did I feel it was a bind”*  **PtW2**: “*I could work around that. I used to try and get along on my own, so I used to go, sometimes I went three times a week if I had a spare bit of time”*  **PtC10**: “*I think that for me it was set at the right level”* |
|  | Most HCPs felt they had sufficient time to provide exercise support in consultations as this was embedded into their routine discussions. Additional time was required to attend professional training and complete associated research processes. | **HCPC1**: “*Well obviously I’ve had to attend the training meetings, which for all of us have worked round our clinical commitments, which was really good. As regarding screening patients and things, quite often what happens is they come to me anyway for their first hormone injection. So that tends to be when I screen them, and if they’re eligible then I’ll talk to them about the trial. So, it’s not as if I’m having to book them into different clinics or fit them in here there and everywhere, because I do it as part and parcel of my injection clinic. So, it’s not really been restrictive on my time. And it doesn’t really add anything more onto the consultation, because I’ve got all the things with me. And before the trial I’d always advocate and advise patients to be fit and active and things.”*  **HCPC1**: “W*hen we initially see the patients to start them on the hormone treatment, I’m talking through the possible side effects with them, so I’m already mentioning to them, you know, and to counteract these they need to be fit and active, so yeah.”*  **HCPS1**: “*I will be honest and say that there have been challenges, specifically time. It’s time that it takes in the consultation. And also the time commitment, because I try to ensure that all our team went to the training and that we were all involved so that it wasn’t just one or two members of the team that would be involved in the STAMINA project, we wanted everybody to be able to see the patients and be able to talk to them about it, because we work very much as a team rather than as individuals.”*  **HCPS1**: “*That’s probably a little bit more cumbersome [screening log] and I have to be honest it’s probably the thing that we’ve been the least proactive with. Just simply because you just, it’s time, and I know it sounds like an excuse but we are just incredibly busy, and anything that adds to the paperwork and whatever does slow you down.”*  **HCPC2**: “*So, my involvement, so I’ve been to the training. And I have, I think I’ve drawn attention to it to my colleagues. And at the moment there’s a big focus on prostate cancer at this trust. And there is nationally but at this trust. And so I’ve been asked to give lots of presentations at* *various places. So, part of my role has been also really just telling people what we’re doing. And even just being involved in the trial has been really helpful, even at just this stage.”*  **HCPC2**: “*My role has been really, because a lot of what I do is giving men their diagnosis. My role really is of signposting it. It’s very hard to go into the trial in any great detail when you’ve just told a man he’s got cancer and he needs hormone treatment. So how I play it is I’ll give the diagnosis, explain the treatment, explain fatigue is a side effect of hormone treatment, and I will mention to them that exercise is good for helping the fatigue and muscle mass and bone etc. from hormone treatment. And I’ll explain to them that we are running a trial of supervised exercise. And I’ll kind of leave it there and just say XXX will talk to you more about that, because at that point they’ve had a huge amount of information. It’s not appropriate for me to go into it anymore. Really I just need to sow the seed.”*  **HCPS2**: “*I find it relatively easy because I have a discussion about diet with patients, how they can modify their diet, so exercise goes hand-in-hand with those patients. So, it’s relatively easy to have a chat about the importance of exercise…. I think it arises very well and I think it’s twofold. Because once I’ve had the discussion with the patients and put the bee in the bonnet, when I leave the CNSs take over and they reinforce that. So, it works well. I think they get, the patients get two hits of the STAMINA.”*  **HCPC3**: “*I don’t what you’d officially call me, a recruiter I suppose would be the main thing. So, my main role there would be to identify patients who are suitable for the trial. So, men going on to ADT with advanced prostate cancer, but not about to have chemotherapy. So usually I just recommend the trial to them. So far I think I’ve recruited one patient. One would have been recruited yesterday, but I was told we’re full. And really just to recommend to them it’s generally a good idea, whether it’s good or not for prostate cancer and being on ADT having a healthy exercise programme, so positive attitude for it. And after that I don’t have anything to do with it really … It’s easy, because I don’t have to do much.”*  **HCPC3**: “I*t didn’t make any significant, I’ve not had to change my clinic timings, I’ve not been running late. But then again, I’ve only seen three patients that were suitable.”*  ***HCPW1****: “So, we identified men from clinic and there’s another clinical oncologist who’s there as well and I think he identified a couple as well. And because we were being very enthusiastic about it, you know, we were concentrating very much in that short period of time on the study, XXX is quite a small hospital actually because we don’t have a huge number of completing studies, and we certainly identified the patients, spoke to them about the potential to go into the study, checked their eligibility to make sure they didn’t have any uncontrolled cardiovascular problems or medical reasons why they couldn’t do the exercise. And then the research team would come and give them the pack, which the research nurse, which they would take off and read and then the follow-up call was from XXX STAMINA team…. So it was really about identifying and explaining to them what it was and that there’s evidence behind supervised exercise, trying to improve quality of life from just fatigue levels and just making sure that they were actually medically fit enough to go, to end up where the majority of them were.”*  **HCPW2**: “*We identified patients that could go forward obviously because we were hands-on with them and I did do one of the recorded discussions.”*  **HCPW2**: “*It depends what would be required when it rolls out. If there was a huge amount of paperwork to fill in, i.e. it would be not necessarily done by the trials nurses but if it was expected to be done by the clinical nurse specialists at the time of our consultation that might be a bit tricky. I think we’d still need input from clinical trials teams. But in terms of thinking about patients who are eligible or not eligible that is just part of general assessment of the patient isn’t it? So, I don’t think it adds a huge amount as it stands, providing you still have the backup of trials nurses if that makes sense.”*  **HCPW3**: “*Yeah so we obviously pre-piloted back in October. We were identifying the patients in various different ways. The way we ended up getting the patients was via the consultant clinics. So, it wasn’t quite recruiting directly I guess because I think we ended up getting them through the consultant clinics instead. But we were definitely able to identify patients that would be suggested as being suitable for the study.”* |
|  | EPs had protected time to deliver the STAMINA intervention and were able to schedule and supervise exercise sessions with ease, on and off shift. Additional time was required to attend professional training. | **EPW1**: “*My job would then be to basically contact the men, the patients themselves, get their understanding of what the STAMINA programme was, and essentially book them in for their first session with one of the PTs. And obviously manage their expectations on what that first session would look like and make sure that they were prepared and had the right kit and knew what to expect really because for a few of them it was a little bit intimidating, so.”*  **EPS1**: “*So, I do their initial tour of the facilities, set them up a membership and then discuss with them about which member of staff would be ideal for them to go. So, it’s more, for me it was very much my usual role. So that was quite good.”*  **EPS1**: “*So, I went on the same training day that my team went on. So, I know exactly what they have been through training-wise, which I felt was important. Because then it meant that I could support them if they had any questions, so I understood what they were delivering. But, yeah, apart from that it’s not really kind of, not much extra time. It’s a 10-15 minute tour with* *somebody talking to them depending on how much they want to talk, and then just setting up a membership can take us five minutes or so*.”  **EPC/S2**: “*I’m the first point of contact for every STAMINA client that comes through. So, I’m the first person they’ll meet. So, as they come through from Nuffield Health. … So, I’ll greet them once they get to Nuffield. I’ll show them round the building, they’re going to take part in XXX or XXX myself. And then it’s conducting, checking medical history and checking everything from the referral that I’ve received from the university team. Just discussing that through with the client, making sure they are able to exercise.”*  **EPS3**: “*It’s been good. It’s been easy to deliver because it’s just a PT session really isn’t it? It’s quite easy because you’re going on - it’s a bit different where you sort of just, the cardio is quite different from what you’d usually do, because usually you wouldn’t sit for half an hour, 40 minutes with someone who was paying you for an hour PT session. So that’s a bit different. So sometimes that feels a bit weird just to be sat next to them. But yeah, it’s been fine. Most of them are quite able”*  **EPS3**: “*Off shift [delivering] because, well I’ve had a couple on shift, a couple off shift. It depends the way our shifts fall to what days. Most of them are stuck with the same dates. So, once you’ve got a set pattern you’ve got that. So, it just depends if your shift fell, if your shift moves around, you’ve ended up on shift if your shift’s. So yeah, because you’re treating them as a PT client as well really, because you need to block an hour out for them each time; it all depended on when your shift fell…. but it’s just a lot of them want early mornings. Well not early mornings but like nine, ten, eleven. So, unless you’re doing a morning shift, it’s always pre-shift.”*  **EPC3**: “*No. Been really easy actually to get them all booked in, so they’ve all been kind of, if there’s ever a time that they’re on holiday or anything we’ve just worked around it and tried to change the date for them, if possible. But other than that, yeah I think it’s been very easy actually. A lot easier than I thought it was going to be.”* |
| 1. **Ethicality**   (The extent to which the intervention has good fit with an individuals value system) | The men varied in their attitudes to exercise and some expressed initial apprehension when the intervention was introduced to them by HCPs as, for example, attending a gym was outside their realm of experience. However, when the potential benefits of the intervention were explained to the men, they reported feeling positive about attending, that it would fit into their daily life and that they were likely to benefit from participating. | **PtC4**: “*When I was diagnosed originally, no I’d never give exercise a thought. If you’d said to me six month ago you’d be going to the gym I’d have laughed at you … but as Dr XXX told me, it’s manageable.”*  **PTS5**: “*I was apprehensive cos it must be 20 years since I went to a gym … but once I got there, it was good.”*  **PTC12**: “*I have a friend who has gone through the same thing with prostate cancer and when he was on the hormone treatment he did tend to put on quite a bit of weight he said to me. So, one of my thoughts was if I went on this programme at least if I didn’t lose weight or whatever, if I could maintain my weight then that would be very useful to me, yes.”*  **PtW2**: “*I was a complete couch potato. Never really took any exercise at all.”* |
|  | The intervention aligned with HCPs professional development goals and beliefs about the benefits of exercise for men on ADT and the importance of embedding exercise into the prostate cancer care pathway. | **HCPC1**: “*Very good yeah. Obviously if I’d never done research before I’d be developing research skills, but obviously I do do research; but just being a part of big trials is always good for development.”*  **HCPS1**: “*We’ve found it really interesting on the first level. I think we all feel we’ve really benefited from it, because we’ve learned through it. I think we all really believe in it, because it’s an area that has been lacking for patients in terms of information and advice, but also resource. There’s been nothing to offer patients in terms of exercise and that kind of thing, which is difficult when you’re telling somebody that that’s what they need to do, but there’s no facility to provide that.”*  **HCPS1**: “*I think what we actually found was that being able to offer it was almost a relief, because it meant that you could do something constructive and something positive. You then felt guilty when you had patients who either weren’t eligible or once we stopped recruiting, because by that time we’d got into the flow of things, and it was normal practice. And then you were still having to talk about the importance of exercise and the NICE guidelines, but we’re back to having nothing to offer them.”*  **HCPS1**: “*I think integrating it into your normal consultation is really important. I think when you’ve just diagnosed you see lots of different people, and I think when you’re then saying and I’m going to hand you over to somebody else, I actually really enjoyed it. I think I can speak for the team, we all felt it was quite a natural thing to do. So, it didn’t feel as if we shouldn’t be doing this and there should be somebody else doing it, it is just the time factors, and I think that does need to be addressed.”*  **HCPS1**: “*I think it gave us an opportunity to offer something that didn’t previously exist. So professionally that feels the right thing; that feels better. I think it’s really difficult when you know that somebody should be doing something, but there’s no provision for it. And talking to XXXX prior to the study he was saying to us in a way it’s a pity, you almost want to be able to prescribe it for somebody, to prescribe the exercise along with the MDT. We do all of that, we’re very medical, but in terms of the more holistic approach and what patients can do. And patients want to take some responsibility, they want to feel involved…. You know, to sell something really strongly, but not be able to deliver what it is they need is really quite difficult. Because it’s like saying you can have this bit on the NHS, but then you’re going to have to pay for this bit privately, and that feels really uncomfortable.”*  **HCPC2**: “*So, part of my role has been also really just telling people what we’re doing. And even just being involved in the trial has been really helpful, even at just this stage. Because in talking about the trial and drawing attention to the fact that there isn’t a supervised exercise programme for our patients when there should be, as per NICE guidelines, actually it’s meant that the people at the Macmillan information centre, they’ve had meetings with in community. And we’re trying to get, as part of the Live Life Better XXX programme we’re trying to get cancer back on as part of supervised exercise programme, because at the moment, men who have cancer can’t access it. So, they can access it, for cardiac disease or obesity, supervised exercise, but they can’t for cancer. So, they’re trying to get that changed as part of that programme … So actually the fact that I’ve been promoting what we’ve been doing with STAMINA has actually had an impact in the community. And if we can get as part of that programme, you know, cancer back on as being able to be a referral for supervised exercise programme, we’ve done something really good there, outside of the trial but for local men.”*  **HCPC2**: “*The problem has been that there’s been no way to refer patients. And the more you can do is just advise them, exercise and keeping active will help, I know you’ll feel fatigued from hormone treatments but exercise and activity will help. You know, when you’ve got nowhere to refer the patient to, it’s difficult really to go further than that, so yeah.”*  **HCPW1**: “*I think the main thing about it, I was quite surprised by how well they did on the programme, because there were a lot of them that did have comorbidities, and I thought that would potentially mean they either didn’t attend, although the guy with gout just couldn’t attend in the end because of that. That they wouldn’t attend or that they wouldn’t be able to complete the programme, so I’m quite surprised at how well they did and the fact that they all said yes.”*  **HPCW2**: “*To date, prior to STAMINA what we had was our energise programme. And it was always something that we would talk to the patients about if they seemed keen on doing something about their weight gain or just general lack of oomph. So, we didn’t push it for everybody.”*  **HCPW2**: “*I think it would be extremely helpful to men. My reservation is it’s going to be very difficult to fund and keep going. I think it’s a great idea but like a lot of great ideas it’s costly. And I’m not sure whether in the current climate that’s going to be sustainable and that would be a shame”*  **HCPW2**: “*Yeah it fits very well actually. Because we’re right on the coalface if you like, seeing patients starting them off on their hormone therapies, talking through their, the guys with metastatic disease are talking about their futures, everything from diagnosis really so we’re right up there.”*  **HCPW3**: “*Absolutely and those were the kind of patients we actually were surprised by but I think do benefit from it more because they’re not your general fit and able and they have got diabetes and they’ve got other health problems but actually saying exercise is beneficial and picking these patients not just the patients that come in and having a chat about exercise has been interesting and I guess an eye opener for all of us really.”* |
|  | The intervention provided EPs the opportunity to help people and have a positive impact on peoples lives which aligns to their individual and company (i.e. Nuffield Health) values. Furthermore, the intervention supported EPs professional development goals. | **EPC1**: “I*t’s another bow to our string, but also especially with our background at Nuffield, with the clinical side that we deliver anyway we have the experience and it makes it more interesting as well.”*  **EPW1**: “*Yes definitely. It was really nice for me, I’ve been in the industry nearly 15 years and I’ve not yet dealt with, this is the first time I’ve dealt with that kind of programme. I’ve trained cancer patients before on a one-to-one basis, but never in a group setting and certainly not as part of a larger programme. So, for me it was a great experience and the gentlemen that we’ve had in the programme have been absolutely lovely as well.”*  **EPS1**: “*As my role as fitness manager it’s just really nice to have a really positive project to be part of and to give my team, so it’s nice to give them something that they are going to feel good about, that they’re going to get a benefit from in terms of being upskilled and that sort of thing, and having that experience. But it also means that we can attract a better calibre of PTs, because you’ll get people that are interested in doing these, and being part of the project and therefore wanting to work for Nuffield, which is nice.”*  **EPC/S2**: “*So yeah, it’s been really quite good actually to spend a bit more time with the patients and hopefully positively influence their life going forward in terms of knowledge and dealing with the prostate cancer.”*  **EPS3**: “*So sometimes that feels a bit weird just to be sat next to them. But yeah, it’s been fine. Most of them are quite able. So, there’s a lot sort of, obviously XXX has to highlight injuries and stuff like that, but there are lots of more capable than what they think at first …. Yeah, a bit frailer than what they are. Most of them are pretty tough. Especially around here like, most of them come from quite a manual labour. It’s coal miners and metal workers isn’t it around here, so they’ve always done hard jobs. So, they’ve been pretty tough. They’ve got a good base level of fitness.”*  **EPS3**: “*So obviously you’ve got the recording of all their information and stuff like that. And you just build a bond with them don’t you really? You get to know them. Two hours a week is quite a long time. You see them more than what you’d see your friends and stuff like that really. So, you spend a lot of time. So, you get to know them quite well, which is quite nice. You learn about them and stuff like that. And a lot of them are very happy to share their experiences and stuff like that, so it’s quite interesting. And yeah it’s good development as well, because it’s good to say that you’ve been involved in the project, so it’s good career development as well.”*  **EPS3**: “*Being a charity [Nuffield] it would be nice to think that they would offer packages for it. But it’s, it would be good to push towards the council gyms and stuff, because they are in every area; whereas Nuffield’s aren’t in every area.”*  **EPS3**: “*For me it’s quite good because I swapped to doing personal training because I wanted to work with medical and rehabilitation. So, it’s quite good for me personally because it’s a good thing on the CV, isn’t it, to say I’ve worked with this. So, progression for me is quite good. Other people will just, it’s just personal isn’t it, on how people want to progress. So some people do it just because it’s part of working for the company, but some people volunteer to do it because that’s the career path they want to follow, and for me like medical stuff is the bits that I want go onto.”*  **EPS3**: “*All right, it’s different for me, being honest. My role with my clients is we don’t go on the cardio machines because they can do that in their own time. So that was probably the first time in my two years of being here that I’ve actually got people on the bikes, just the standard bikes and stuff like that. We use the rotors and stuff, but treadmills and bikes and stuff I don’t usually use. So that was quite good. But it gave me a good kind of, like I say, platform to get to know them and talk to them and stuff. So, they’re not just coming to the gym to sit in silence;* they’re *coming to have a chat and that. And we have some conversations about all sorts now like, I don’t know, loads of things. So, it’s been quite good actually, kind of having chats with them and stuff and getting to know them. So yeah, I’ve adapted to it all right.”*  **EPC3**: “*I suppose when you see that initial sheet, certain things like avoid treadmill and things like that because of certain things, before you’ve even met them you start thinking are they going to be really frail? And they’re going to be really delicate and then it turns out they’re not. So, I think whether or not we need to communicate that a bit better or. I guess you work it out anyway when you meet them anyway. When they start chatting about what happened and stuff…… So, it’s been quite good. I feel like I’ve had an impact on their lives a little bit which is nice. So, it’s good.”*  **EPC3**: “*Definitely, in this kind of gym anyway yeah, couldn’t imagine it being done in other gyms, but yeah through Nuffield, because we’re more of a health club not a typical gym…. Meathead gyms, dog eat dogs, trainers just in there because they want to look good and they want to get money; whereas here we actually like helping people. And I suppose that is a prejudice sentence, but it is more what Nuffield are about isn’t it, like we’re all kind of, we get to be part of things like this, STAMINA and like other things that we’ve done. We are here to help people. So yeah, I think it’s definitely the right kind of gym to do it. And the right range of trainers as well, so yeah. But then again, you don’t know what other gyms are going to be like so.”* |
| 1. **Intervention Coherence**   (The extent to which individuals understand the intervention and how it works) | Men generally demonstrated a good understanding of the intended pathway to benefit of the intervention. They reported that it targets both the physical and psychological consequences of ADT treatment with some believing that it could also help them recover some control, which a cancer diagnosis can take away. | **PtC4**: “*There’s some chaps in here, not to be disrespectful, they’re well overweight. But they’re here, they’ve come and they’re trying to do something about it. They’re not sat in a corner thinking oh that’s no good for me.”*  **PtS5**: “*If you go along with the right attitude and you’re prepared to put in as much effort as you people in getting it up together, you’ll get a lot out of it.”*  **PtC12**: “*I did yes because I have a friend who has gone through the same thing with prostate cancer and when he was on the hormone treatment he did tend to put on quite a bit of weight he said to me. So, one of my thoughts was if I went on this programme at least if I didn’t lose weight or whatever, if I could maintain my weight then that would be very useful to me, yes.”*  **PtW2**: “*I was certainly looking forward to it, it’s something I wanted to try. How effective it would be, I didn’t really know, but certainly it’s something I wanted to try.”* |
|  | HCPs demonstrated a good understanding of the intervention components and its intended outcomes, i.e. the positive impact of supervised aerobic and resistance training on the physical functioning, psychological health and social wellbeing of men on ADT for PCa. More specifically, how exercise may offset the side effects of treatment. | **HCPC1**: “*Well it gives them a structure, and I think it, in some respects if they’re doing exercise, and they know it’s going to help them, I think psychologically it will be of benefit to them. As well as physically, because there is the odd man that I’ve got on the trial that is a bit portly if you like, so it might help them lose weight, which is good. So yeah, and I think if they can get within a group or when they’re going for the sessions if there’s other men there that have been because of the trial, the camaraderie.”*  **HCPS1**: “*I think it gives them something positive. I think it allows them to take control. I think it helps them, obviously from a side effect point of view we hope the benefits will speak for themselves, but I think men quite like to be proactive. And I think the fact that they have got opportunity there was really well received by them. There were some who were never going to be interested, and that would be the case regardless of whether STAMINA was available to them or not. But it made us, having that conversation about the importance of exercise a lot easier when you then had, and there’s an opportunity to do this …. And not everybody has access, not everybody can afford gym membership. Given that the NICE guidelines specify supervised exercise, that’s not possible for many people for lots of reasons. But I think this opportunity was really well received.”*  **HCPC2**: “*So, I think psychologically it’ll be good for them. I think physiologically it will be good for them. I think they’ll be loads of health benefits for them, mentally and physically. I mean they’ll feel better about themselves. They’ll feel, it will improve, so muscle mass, bone strength, they’ll be all sorts of ways it’ll be useful.”*  **HCPS2:** “*Yes, I think so. Where I mentioned diet is it’s all good and well talking about treatment for prostate cancer, but you’ve got to empower the patient to look after their health and general wellbeing. So, exercise and diet are both the [unclear 0:05:29] factors. And it’s surprising how many patients don’t know, men, their families don’t know the importance of exercise and the wellbeing; particularly with the potential long-term side effects of certain treatments of prostate cancer.”*  **HCPC3**: “*I can’t imagine why saying to someone you might need a bit, have a free year at Nuffield gym if you want and do some exercise. It’s not, there’s no, it’s not like trying to get someone to consent for their organs for a dying baby. It’s just, which I have done in the past jobs, it’s just nothing is it? It’s just do you want to do some exercise this might be a good thing for you. It’s easy. And it takes their mind off the cancer thing as well. It’s a positive thing that you can talk about, other than sorry mate you’ve got cancer. So, it’s no problem at all.”*  **HCPW2**: “*Because there was some research behind it and the general feel for there’s something new come in that we could look at that could maybe help you, they were all oh yes that sounds like a really, I’ll be interested. [Unclear 0:04:37] spoke to one of my follow-up patients who is actually on the programme just the other day and he’s found it an incredibly positive experience. But he was a man that used to play squash regularly anyhow. He’s one of the old chaps who was actually extremely fit for his age, missing that in a way, so it just sort of, come as a different dimension to him. He feels that he doesn’t get pushed as hard as he would like to be pushed. But I explained to him that we weren’t trying to improve his fitness per say we were just trying to give him this extra dimension of care in terms of managing fatigue and all this. You can see the worth of it. He’s still really keen on it.”*  **HCPW2**: “*I know that I was very biased in terms of I thought if they were pretty fit they might want to do it but I certainly wouldn’t have put somebody who was perhaps had difficulty in walking forward for exercise. I thought perhaps if you went once around the garden that would be pretty good going but now they’re suggesting that they do something a lot more structured than that. So we’ve always had exercising in the back of our head for bone health and muscle health, but now obviously this extra information about the absolute benefits of the structured exercise programme where you’ve got your different types of exercise, it’s sort of changed our way of thinking. And now I know I can’t forget what I now know, so I think my conversations with patients who don’t go onto STAMINA but are going onto hormone therapy are different now.”*  **HCPW3**: “*I think prior to the study we were very much waiting for the conversation to come up rather than being forthcoming in having that conversation. I think after the study if someone is suggesting side effects of fatigue and muscle mass, there’s much more of an open door about having that conversation even without a prescribed exercise programme being offered. We have got an element that they can go on to exercise programmes but not quite as robust as STAMINA, I guess, but also just giving the reasoning and the NICE guidance and suggesting that this is beneficial and actually having some evidence base I guess around it and just appreciating that it would be beneficial for them is enough for us to have that conversation with them. Definitely in terms of our nurse-led clinics I think we probably are in the best place to have those conversations in terms of patients coming up with follow up at five years or two years, three years and all the rest of it and have got fatigue. So, I think not just fatigue but the other side effects. It’s definitely worth it.”*  **HCPW3**: “*But I think definitely in the way it was delivered in terms of it’s a prescription given by your oncologist, we recommend that you do this because of these side effects and actually them being able to choose to do something or do something for their prostate cancer which there isn’t much in terms of, well apart from smoking and normal lifestyle changes, actually being told this is what you can do and it will help you definitely put a bit of self-management on them. So, there’s a good element of them being able to do something for themselves which I guess quite often we take that away from them as healthcare professionals. So, it was nice to see that they’ve come in especially with a bit more confidence and yeah.”* |
|  | EPs expressed a good understanding of the positive impact of supervised aerobic and resistance training on the physical functioning, psychological health and wellbeing of men on ADT for PCa. | **EPC1**: “*It’s normally between three to five at a time, with one trainer, and the good thing is, so they’re making friends as well, so they’re not isolated, and I think they stop and realise it’s not just them that’s going through the same symptoms, all the problems they’re having; in fact we’re finding they’re meeting other people with the same symptoms and the same diagnosis, they’re actually a bit more accepting the exercise.”*  **EPW1**: “*I’m pretty sure everyone we’ve seen has reported feeling more awake, energetic and fitter and stronger on a daily basis as well. Even if it’s only been day-to-day things, being able to do some DIY easier or go for their morning walk and walk for a little bit further and a little bit longer. They’ve all reported positive results*.”  **EPS1**: “*Well I think it should hopefully give them some more positivity towards their situation, which is always important for recovery. And then also because they are exercising they will get a bit of physical benefit from that as well.”*  **EPC/S2**: “*Yeah one hundred percent, so we’ve seen cardiovascular improvements, strength improvements. I’m getting people coming to me going I’ve got a bit more energy now: I feel like I’ve got more of a purpose, a bit more of a focus and it’s something I look forward to and enjoy doing. So it leaves people that might not have been working anymore, who’ve now retired, have not got an awful lot going other than hospital appointments unfortunately, it’s given them something that they actually look forward to doing and get enjoyment out of.”*  **EPS3**: “*I know the first guy that I had, he’s been, I think he’s done stuff with you guys for the local news and stuff like that. So it shows that it is working. And as long as you can get people moving in any form it’s good isn’t it? So the longer that they can make their lives easier; it’s important isn’t it really? They don’t, it’s not like, I think someone said when we did the training they die with it not from it. So the guy that’s, the one that emailed back that said that he couldn’t do anything, today he sat on a bike. So the improvement’s there. He might not be doing as much as other people but for him he’s now standing better without his stick. But these guys are the people that probably couldn’t afford personal training, or they couldn’t afford a gym membership, so helping them for the year.”*  **EPS3**: “*Because that’s what you want isn’t it, you want to improve people. …. So it’s definitely improving their lives, just how much by each person is weighed up in their small achievements isn’t it?”* |
| 1. **Opportunity Costs**   (The extent to which benefits, profits or values must be given up to engage in the intervention) | The men felt that there were no notable opportunity costs related to engagement with the intervention, and that making the time to attend sessions was worth the commitment. The majority were retired but some attending the gym whilst in full time employment. | **PtS1**: “*If I can’t find an hour and a half of my day to come and do this which is extending my life, that’s the way I look at it, I’d be a fool. I really would.”*  **PtS5**: “*Arranged around hospital appointments.”*  **PtC10**: “*It does make a mess of life [scheduling holiday etc]”*  **PtS1**: “*Because as I say you’ve other things to do in life, and I’ve also got, well you’ll be aware of it, I’ve got to go through the radiotherapy, and that will take a couple of hours or more.”*  **PtS7**: “*Sessions were arranged to fit in with caring responsibilities.”*  **PtW2**: “*It worked out for me because the 3 months with the trainer ended just as my radiotherapy began. So, I’m doing the radiotherapy now, and I think I would find it difficult juggling the times*.”  **PtW2**: “*Well going on holiday, going to events, weddings, funerals all that sort of stuff … I’m sure you can work around it …that would allow people then to accommodate anything else that happened to turn up in the meantime. if the programme is slightly more flexible.”*  **PtC12**: “*Being retired it’s not too difficult. We tried to arrange the times to suit me because mainly in the morning which was very helpful to me which they did all the way through the programme because I was actually going for my treatment, my radiotherapy in the afternoon.”* |
|  | Intervention delivery aligned with HCP usual practices however some changes were made to optimise intervention delivery; these were deemed non-problematic. For example, rescheduling patient appointments to attend training or to discuss study details within the allocated recruitment window. | **HCPC1**: “*If it becomes a bigger study, and perhaps the inclusion criteria changes, as in if they decide that they can have people on any length of term of hormones, you know, there’ll be more people to talk to, but I’d still do it within that same clinic. Occasionally when I’m at a new diagnosis, if they start them on hormones, we’ll do it as part of that discussion if we know that they’re going to be on them for more than a year. So it’s yeah, it’s not really impacted at all.”*  **HCPS**1: “*So, two of us went one day and the other three went on a different day. But finding time when you can be away from clinical activity to embark on that is actually quite a challenge in itself. So that was an element that was supported thankfully. We had support from our management team, which made that possible. But then it’s not just that aspect of the time involved; it’s the time then when you’re spending longer with each patient to go through the study. And then initially when we were recording all the consultations, without a doubt that does take more time. And then the referrals over to let you all know that the patient had expressed an interest, that we’d done a recording etc. Not huge, and I think the more you do* *the slicker you get at that, but initially, obviously this was only the pilot phase, and it is difficult because you’re just not all quite that comfortable.”*  **HCPC2**: “*Yeah. It’s quite a big commitment [training] especially when you’ve got, you know, if you think about having all your consultants there, you know, that’s quite a lot of consultant time. So yeah I think it might benefit from being shorter with some e-learning perhaps ….. we should be talking about exercise to men who are on hormone treatment anyway. And certainly if we get via the Live Life Better Derbyshire programme, you know, I would expect in the future, either for those men who are not suitable for the STAMINA trial or because maybe they’re out of the year of hormone treatments, or when the STAMINA trial is finished, I would expect that I’d be discussing it with patients anyway.”*  **HCPS2**: “*No, not really because I mean when you see patients the first time, sometimes you have to see them the second time to discuss the treatment options. But when you see them and you’re breaking the diagnosis of cancer, you’ve got to have time for those patients. So, I think it’s important that if it’s an extra few minutes to talk about the STAMINA, it’s not really a major impact on the clinic.”*  **HCPC3**: “*I’ve been to two training days or there was a site induction day and a training day, and got lots of information about the trial…. Well there was no effort other than time, if you know what I mean. So, it was four hours wasn’t it, a couple of hours on two Fridays. So no, it was no effort really, as long as you’ve got a couple of hours to spare on a Friday afternoon ….. I don’t think you’ll find all urologists across all the sites prepared to give up a whole afternoon of their time to train. Maybe you will, which would be great if they did. But I think there’ll be some people who can’t, won’t, don’t want to. But they could still recruit people without all that training quite easily.”*  **HCPW1**: *“It did take a bit more time, because it’s not normally embedded in our practice. So, we were spending a bit more time talking to them about exercise and the study, but then research isn’t daily part of the oncology clinic.* *So, if we’re introducing a radiotherapy trial or a drug trial then we will spend more time in clinic talking to them about it. So, in a way it was no different and the fact the end point is possibly more satisfying for them.”*  **HCPW2**: “*Certainly, I think once people got the gist of it, it was one of those conversations that then flowed, because you have the information to back you up. It’s not like you’re floundering around. You’re saying this is what we could offer you. This is what we’re going to do. Obviously the patients I see now in follow-up clinics generally who happened to be on STAMINA, it’s just one or two little sentences about how you’re doing, how did you find it, are you liking it, are you* *not liking it, are you still doing it, those sorts of things. But that’s about it. It’s not particularly deep. It doesn’t take much longer.”*  **HCPW2**: “*Yeah that seemed to be OK because we all managed to attend it [training]. We have quite a schedule with the clinics and we were able to adjust those accordingly so that worked well for us.”*  **HCPW3**: “*So, recruitment-wise I think it was a bit different because of the pre-pilot and us knowing we had a timeframe on having to recruit the patients. So, I think we had a 12-week area of having to recruit up to 10 patients. So, we were quite aware that we needed to identify patients. I think in a normal day-to-day basis we would have been able to recruit but I think due to the elements of the study, things like having to record the patients, we were slightly working in a different way, having to bring appointments forward which again isn’t the most ideal thing, but for the sake of the study it was the most appropriate thing to be able to get them in.”*  **HCPW3**: “*It was quite difficult just in terms of us all having to stop clinic [to attend training] so I guess appreciating that we all run nurse-led clinics and consultant clinics and trying to identify a time available for that really. We managed to do it and we just had to sort of shut shop and come to you guys but I think given enough notice it’s reasonable to do and especially when we know the benefit is for the patient and actually being part of the study was feasible in the long run anyway.”* |
|  | Most of the intervention aligned with EPs usual practices at Nuffield Health. For example, protected time was provided to attend training. However, some changes were made to optimise intervention delivery. For example, rescheduling the physiologist’s clinical commitments or working longer hours to deliver the intervention ‘off shift,’ however there was a preference for this. | **EPW1**: “*Well I actually was [delivering sessions] in the end, that wasn’t the initial plan, initially what was going to happen is I would sort of coordinate it and the PTs would be actually delivering the sessions, and my job would be then to basically manage the payroll side of it, but also feed back to the STAMINA team any issues and update them on the progress of the men with the PTs; unfortunately we had a couple of PTs who dropped out of the programme for various personal reasons. One was leaving the business anyway. So, I did end up delivering probably half of the sessions that we’ve done.”*  **EPW1**: “*For the training itself, yeah that was absolutely fine, yeah it wasn’t an issue. Yeah at site when we’ve got programmes like that the way Nuffield tend to work is when you’ve got programmes like that, if there’s training that you’re required to go on time is made for you to do that, so.”*  **EPW1**: “*For myself, it has meant some longer days. So if I’ve been on, because I do teaching manager shifts as well in my role, so if I’m down to do a late shift which is 2pm until 10pm, if I had STAMINA patients in the morning that might end up me working 10am until 10pm that day. So, it has been some long days, but in terms of delivering the programme itself, yeah it’s been absolutely fine.”*  **EPS1**: “*I mean we’ve got set days when they will be coming in to do their first assessment. So, XXX will let me know in advance if someone is coming in on the Tuesday for their assessment. And then I know that I will need to meet him after the allotted hour to then take the person for a tour around and do all of that. So yeah it works well*.”  **EPC/S2**: “*In terms of the training it’s a little bit more difficult in terms of, because obviously what we have to do, we have obviously days off diary. So, what I mean by that with a personal trainer typically on a rota shift pattern; whereas a physiologist will be working across regions. The way the physiology team is structured it’s regional … So, the training’s been good, but the issue that we’ve had with this sometimes in terms of fitting it around screening commitments. Because obviously as a physiologist I’m needed within the region to see clients, sometimes it’s been a little bit more difficult trying to get me out of those commitments due to capacity issues really.”*  **EPS3**: “*I thought that a lot of it [training] could be, you could get away with having like a half day and then doing the online stuff. With time it just frees it up a bit… It’s just because we had to do all of that and then go for the day. I mean sometimes when you’re only doing, our shifts are only, we only have 16-hour shifts, a full day’s training takes you out the gym, takes you off the gym floor for, and that’s contact time with trying to get clients. So, I think it’s good to have all that training, but then on the flipside you lose a day on trying to get other stuff. So it’s good to be involved in it all, but slowly your days get filled up with more training and you have less time.”*  **EPC3**: “*Fine, because they were all kind of happy to do the days that I have the space basically. So Tuesdays and Thursdays and they all had these, they were all pretty free on those times. The only times it would interfere was if I had to be on shift. So, if I was on shift and we’ve got one booked in I’d have to kind of either rearrange or make sure I was blocked out and able to do it. That’s the only downfall. But when shift work comes into it, but doing it off shift has been a lot more, a lot easier. Yeah, I think I’ve only done one on shift so far. Again because of the shifts and holidays and stuff always changing that would be difficult trying to get them in and then making sure. Because if you had no space on a certain week because everybody’s booked you in for MOTs and stuff then you’d be struggling, so being able to do them off shift has been very beneficial, definitely.”* |
| 1. **Perceived Effectiveness**   (The extent to which the intervention is perceived to be likely to achieve its purpose) | The men reported perceived improvement to their physical fitness, including cardiovascular health, and what some termed “*general fitness”.* Many stated that more importantly for them, participating in the gym sessions had resulted in significant improvement in their psychological wellbeing and mood, and that this had given them a more positive outlook, enabling them to be more proactive in their daily life. | **PtS1**: *“I think the physical health yeah. But also, I was surprised, you say mental health, it’s a funny subject really ain’t it but yeah. For instance, I’m doing less of the putting things off.”*  **PtS1**: “*I think the cardio has done quite a lot. And as I said to put a bit of weight on, tighten the skin up, so yeah. I shall be like a 20-year-old.”*  **PtC4**: “*It’s made a complete difference to my life in general, my head, you know, everything. I look forward now to everything.”*  **PtC8**: “*Before I went to the gym [half mile walk] was taking me 40 minutes and now it’s 25 (+ appetite improved and sleeping better).”*  **PtC8**: “*It was brilliant … it took me out of myself.”*  **PtC4**: “*I can feel myself getting fitter”*  **PtS5**: “*It was the mental part more than the physical … Sometimes I was down, I was a bit low, but when I was doing the exercises I did feel more uplifted.”*  **PtS7**: “*I’m going to have to say no I haven’t really seen any improvement. But it’s just as important when I say but I haven’t seen any un-improvement ….. So you might say well why because you don’t seem to have had much benefit from it. But I think that’s to do, if a 55-year-old was offered to join it would do him a lot of good I should think. You know, he’s probably deskbound at his job and not getting a lot of exercise. So, yeah I’d certainly recommend it yeah.”*  **PtW2**: “*It’s worked really well … the trainer said to me that my heart rate had improved.”*  **PtW2**: “*Well it’s just going to make us fitter. Now according to the stuff I’ve read, I’m on these injections as well, and these injections seem to affect people in different ways … so whether or not doing the exercises can counter some of those other effects. I don’t know.”*  **PtC12**: “*I certainly think you do benefit, because if you think about your general fitness … you know if you do exercises like from a sit to stand, well when I started I was just doing that sit to stand, just me effectively, towards the end I was doing the same exercise but I was holding a 12 kilo weight in my hand up against my chest as I did it. So, obviously that increased gradually. To start with I had five kilos and then seven and so on. So, I gradually increased that. So, you can see it does benefit your general fitness and what you can do. And some of the other exercises where I set off doing things like doing a chest press and things like that with five kilos and working up to 20 kilos and that sort of thing through a period of time.”* |
|  | Most HCPs believe that supervised exercise has physical, psychological and social benefits for men on ADT, including counteracting side effects of treatment, in particular a reduction in fatigue and improvements in quality of life, feeling a sense of purpose and self-worth and improvements in energy and enjoyment. These beliefs were shaped by direct feedback from men on ADT who were participating in the STAMINA trial. | **HCPS1**: “*Definitely, the response we’ve had from the patients that were interested was really positive. And I think one of the things for us when you consider the NICE guidelines, you’re* making *a recommendation but there’s nothing to support that recommendation other than the patient’s ability to either afford it or be self-motivated, it’s quite difficult.”*  **HCPS1**: “*I think obviously physical exercise, which we know will hope to counteract some of the side effects of their treatment. But also I think psychologically it will have a huge impact, and socially, because I think men don’t necessarily, I know you can’t generalise and that sounds very sexist, but women with breast cancer have huge support. They have lots of opportunities available to them. But there’s so little available to men, particularly with prostate cancer, and we don’t currently have a support group. And I think the hope would be for me that they would also then meet other men in a similar situation, and then offer the opportunity. They can either have the one-to-one or they can have the group sessions. So I think it can help on a lot of levels, not just on a single level.”*  **HCPS1**: “*In terms of the actual gym side of things, I guess we’re not in a position to know, because it’s not our field of expertise. I think we’d have to get feedback from the PTs and things know whether that was achievable.”*  **HCPC2**: “*So, the men I’ve talked to and I’ve talked to one man this morning who is really enjoying it. You know, they tell me, I mean as with patients who aren’t on hormone treatment, you know, they go along, I don’t really want to go, I’m tired, I don’t want to go, and afterwards they’re getting that buzz. That endorphin buzz, they feel better. So yeah, the man I spoke to this morning is really enjoying it.”*  **HCPS2**: “*So if, with the patients who are on hormone treatment, they obviously have a reduction in the bone mass, have a reduction in the muscle mass. And naturally as you start to weaken and tire out, you’re likely to just become a couch potato, so you won’t have that drive of doing things. So, I think having a programme from the hospital encouraging exercise and being motivator that I think is very useful. Even other treatments, you know, if someone had surgery or someone was to undergo radiotherapy, you need to motivate those patients and to continue because ultimately you want them to continue their quality of life and improve the way of life. So, I think it’s a good opportunity.”*  **HCPS2**: “*Yeah. So I think it’s good and in some ways it’s quite rigid in some aspects because you’ve got to go regularly etc. but for the benefit of the exercise programme, it can’t be just I feel like going to the gym today; because if you do that, including myself, you don’t go. You need that extra push with a regimental programme to benefit properly from the programme. So I don’t know how because I don’t know the results so far. But I don’t know how it impacts the patients directly because they’re, you know, on top of all their hospital clinics appointments and various* *other things that they have to make time to get to the gym and the cost of it, those are the only things that I would think may have an impact.”*  **HCPC3**: “*Well, if you want me to be pedantic about it, if the answer was yes you wouldn’t need to do a trial would you? There is a randomised trial about to happen, is that right, of exercise versus no exercise? … So I can’t tell you if it’s going to be beneficial. Because if the answer was yes it’s beneficial there’d be no need for a trial, we’d just be doing it wouldn’t we? Does that make sense? ….. So yeah, getting men to do exercise is a good thing, but so is getting women to do exercise, and so is getting men with other illnesses to do exercise. Whether or not exercise is particularly beneficial in a man on androgen deprivation therapy is what the trial’s about isn’t it? So I can’t answer that question.”*  **HCPW1**: “*But* *it was just something positive that we could say look we’ve got evidence that actually if you do supervised exercise then that may reduce the fatigue and improve your quality of life.”*  **HCPW1**: “*Definitely, a couple of the people I’ve seen, they’ve just felt so much better in terms of energy levels, it’s keeping them a bit focused as well, so it’s very easy for them to get down about their diagnosis, metastatic [unclear 0:11:07] the fact that they’ve got to be on this treatment, and the fact there’s going to be more treatment in the future, but it’s helped a lot of them. And made them feel, so psychologically and clinically better.”*  **HCPW2**: “*Certainly, it’s giving them a bit of a fuzzy feeling in terms of I am important, people are taking an extra bit of care for me. There is something we can do about this maybe to feel better and it’s giving them a little bit of purpose in terms of well let’s go out and do something different, so just a little bit of a change. Positive thing isn’t it? So I think it’s that. I wonder whether if it was somebody worked out that if you went oil painting or photographing birds whether it would still have the same impact in terms of it’s still something that’s very good for you and seemed to be of benefit therefore we’ll help you do it, I don’t know whether it’s the fact that it’s exercise makes a grand deal of difference but that’s the thing they’re being encouraged to do to help themselves, [unclear 0:12:37] the crux of the matter.”*  **HCPW3**: “*It was almost like a prescription of exercise which I think the patients definitely, well we saw a patient for example in clinic today who is on our radar of being on STAMINA and he definitely said it was useful in terms of his self-worth so I think hearing feedback like that just makes it all worthwhile really doesn’t it?”*  **HCPW3**: “*Yeah so, I think it definitely helped. Like I said we had a lot more patients that were, we would have put into the study had we had the resources to be able to add more into it and patients were so acceptable of wanting to go into the study. I think it was definitely very useful to have that resource. I think going forward if we had that resource a lot of our patients, they weren’t particularly patients that were previously exercising or would have enjoyed exercising or even exercised before, we have quite a different population down here in XXX or so we* *think, that actually benefit from that and they come in walking with a stick and big belly and all the rest of it but they actually were really up for the opportunity that they had and were very grateful that in the end that actually they found it very useful. So, it’s nice to hear the feedback from the patients as well.”*  **HCPW3**: “*Yeah so that was just fresh in my mind because he was there today but he was definitely saying it gave him a sense of purpose, a sense of self-worth, the fact that he had to go to someone he built up a really good relationship with his personal trainer. Unfortunately, our guys didn’t get to do the six weeks in group. I think they just did the 12 weeks.”* |
|  | EPs were confident that supervised exercise has many physical, psychological and social benefits for men on ADT, including development in confidence, enjoyment of exercise, feeling fitter and stronger and having a positive focus in life. These beliefs were shaped by first-hand experience of delivering exercise to men on ADT over a 12-week period and reviewing/ documenting their progress. | **EPC1**: “*Psychologically it gets them out the house doesn’t it, so they’re not isolated. It gets them into an environment with a lot of people, but what they started to realise was their level of exercise can be the same level as a normal member. So, they’re more surprised what they can do and also the social aspect, meeting a group of people with the same condition but they’re working and help each other through.”*  **EPC1**: “*I think they are, because what you’ll see are most of them are a bit older, but I think the youngest we have is 60 years old, so most of them are from 60 upwards with their age. I think it’s just confidence, because they’ve not exercised for so long, we’ve got to think about what they’ve gone through, it’s very dramatic and very, it’s really knocked their confidence. But once you get started then it works brilliant. So we’re not expecting them to be athletes, we’re not expecting them to be doing the same things when they’re 60 years old, we’re going to start on a level and build things up, it’s just building confidence in the end*.”  **EPS1**: “*I do think they seem to have relaxed a lot more since they’ve come in. It’s only if I see them around the gym, but they just seem a lot more comfortable with the environment and they’re a bit chattier with the team, which is always a benefit. And I do think that it would be really beneficial for them….. I always think that exercise is more important mentally than it can be physically. I mean obviously it has some really great physical benefits as well. And it is really important but, yeah, the majority of the time people use exercise for mental health in that it gives them that escape, or it gives them that joy or that sort of thing. So I think that’s probably the most important thing in this, is that they’re seeing some progress of some kind, whether it’s an extreme progress or not, just seeing something is going to give them some positive.”*  **EPC3**: “*And then as soon as like I say they start tailoring things for them, exercises and stuff, they get proper into it and then end up loving trying all this new stuff. Which I think has kept their interest good. I’ve seen a lot of them come in here like on their own now and getting stuck in. So it’s been good. It’s made a good impact on like I say my first two anyway; not had much feedback off the latest one yet so …… The feedback and stuff I’ve got as well like. Helping them with their energy and stuff outside, like just their day-to-day, that’s the feedback I’ve got. Obviously they have their bad days anyway, but all in all yeah I’ve had positive kind of feedback. XXX’s absolutely loving it.”*  **EPC3**: “*They’ve really enjoyed it and it’s helping them. It’s kind of given them something more to do as well, like instead of sitting at home. So, they’ve really enjoyed it I think yeah overall. If you can get them interested and give them something they enjoy out of it, definitely.”*  **EPC3**: “*Fitness-wise, they feel a lot stronger. So XXX, like I say, is just absolutely smashing it. He’s getting a lot stronger. He’s constantly here grafting. I think he’s found that passion back for it again. And it’s given him more to, like I say, it’s given him more to do at home. His wife comes here as well so they can enjoy it together. So, it’s kind of bringing something new into their life instead of the usual routine. So yeah, I think mentally helping them as well, I suppose. Yeah, I think it’s, from what they’ve said as well, the partners or the wives are very in support of it as well. So they’re loving the fact that they’re doing this, it’s keeping them happier, because they’re the ones that struggle the most from what they’ve said with it. Like coming to terms with the cancer and stuff, it’s the wives that get upset the most more than men, and things like that, so it’s giving them kind of, making them happier for them, if that make sense? So it’s kind of having a bit of a knock-on effect through their family and stuff as well, which is nice, positive, as positive as it can be I guess, so yeah”* |
| 1. **Self-efficacy**   (The participants confidence that they can perform the behaviours required to participate in the intervention) | Most men were confident about participating in the STAMINA intervention however some of the men who described themselves as sedentary or lived with comorbidities that they perceived might impact upon their ability to exercise, revealed that they had some initial apprehensions about attending their first gym session. These men quickly became more confident and were reassured by the EPs who delivered a tailored exercise programme suitable for their level of fitness and any existing comorbidities. | **PtC8**: “*I thought I wouldn’t be able to get anything out of it because of my [co-morbidity] but I was wrong.”*  **PtS1**: “*Well to me and from other people, you know, probably negative that gyms were a no-no … but it didn’t turn out like that. I’ve enjoyed coming here, and the guy who’s been with me, XXX, brilliant fellow, XXX as well, so yeah, and encouraged. I think that’s the big point, is you get more encouragement rather than discipline.”*  **PtC4**: “*In the early days, you know, your first session or two, you think, I wonder what he [PT] thinks to me. Does he think I’m up to it.”*  **PtS5**: “*I couldn’t do the rowing machine [co-morbidity] … because I had him [PT] for all the sessions I didn’t have to start explain to him … he knew exactly what I was capable of.”*  **PtS7**: “*Well, given that I’m not all that fit ….. I’ve never not turned up for one of the sessions”*  **PtS7**: “*I don’t think I’ve done a lot. Well I mean for example we’ve only met once a week and we’ve had the odd week where we haven’t managed to meet. And I don’t think he has put me on a resistance thing every time. So, I think the answer is no he hasn’t overdone. And I think, you see I’ve got an arthritic knee, and also I’ve no thigh muscle, that’s why I find it difficult standing up off chairs. So, I think he’s thought best not to overdo that side of things.”*  **PtW2**: “*Oh fine yeah, no problems with anything at all.”*  **PtW2***: “I mean that’s the other part, that if I’ve been to the radiotherapy I don’t particularly feel like going to the gym when I get back. It’s not exhausting but it probably does make you feel that you’re not keen to, well as I say I’ve got a half hour drive as well and that could put you off.”*  **PtC12**: “*I normally go to a local sports centre where I do low impact circuit training probably once a week and I also do quite a lot of walking as well … I was fairly confident yes.”* |
|  | Initially, some HCPs had apprehensions related to the research processes however confidence quickly developed following professional training and experience within their role. Others were confident from the start of the intervention as they strongly believed in the benefits of the exercise programme and were provided with evidence and skills in training to fulfil their role. | **HCPC1**: “*Not very. More confident now yeah, because I’ve got used to doing it…. Yeah, and it’s just getting used to the best way of doing it. So, you try one way, and oh that’s not quite working, and then. So, I think the consultations are longer now and more in-depth than they were at the beginning.”*  **HCPS1**: “*Not very [confident], and we’re all well experienced CNSs, but you feel very much out of your comfort zone. But it doesn’t take long. Once you’ve done the first couple of patients you get into your stride… It was more the processes. We felt that we, because it was something new that we were doing in a clinic that we’d been doing for a very long time in a particular way, when you change practice it always takes a little while to find your feet and know what you’re saying and what you’re doing. And it was the stumbling over the Dictaphones and making sure that that bit was all OK. And I think we all felt, it’s like anything isn’t it, what you do naturally all of the time, as soon as somebody’s watching you, you start stuttering and stammering and you fall over yourself. And you think what on earth am I doing, I’ve been doing this forever and a day. But once again as I say once we got a bit more confident that bit wasn’t so bad. But it’s anything that’s new it just takes a little bit of time. I think the thing that’s most difficult is if you’ve had a break, or we do rotations into clinics and we all work in different clinics. We share the clinics out between us, and if you’d maybe been on holiday, and then you hadn’t done a clinic, and then you were suddenly back in it three or four weeks later, and you suddenly had a STAMINA patient, you’d be thinking oh gosh what do I need to do? I just have to remind myself of the process here. But again I think that will just become, in the longer term that will become easier and more familiar with us.”*  **HCPC2**: “*Reasonably confident, it didn’t seem to be too onerous. You know, it seemed win-win really. The patients can only benefit. And they get personal training and a year’s free membership to the gym. So, you know, if they want to do some exercise, I think it’s great for them.”*  **HCPS2**: “*I felt fine because, you know, I believe in it and as I mentioned so I didn’t personally find it stressful or something, outside of my comfort zone…. Probably a bit better now because there’s been some evidence that we were taught on that course, on that refresher course. So I think a bit more evidence. You know, I wasn’t aware to the level that it was NICE-approved etc. so yeah.”*  **HCPC3**: “*Well I’ve not delivered it, I’ve recruited patients. That’s what I’ve done, I’ve recruited patients for it, confident yeah, completely confident… All I say is this is the programme someone else is going to do it all. I don’t have to do anything about it really in terms of the minutia of what exercises to do.”*  **HCPW1**: “*Well not particularly at the start and that’s why the first training session was helpful and helped us, the way we talk to them about the exercise and everything that’s behind it. So that was what the first training session was about. And then I think we did become more confident but I do have to say, I do hate being recorded. So we did record quite a few of the interviews and then of course typically what happens is you stop the recorder and then all the information that’s important comes out. So we just kept the thing running and then sometimes you need to bring the research team to get the recorder and they weren’t there, it was all the usual things with having recording devices. But yeah, so it was just, yeah it was just, building up experience doing it, but I think the nurses were pretty keen in doing it as well. They saw a lot of them.…. I think we’ve all got a bit more confident in doing it. And that we’ll now actually start talking to people about it on a regular basis, not just in an ad hoc way. But then it’s the difficulty of getting every single person, so yeah you may think it when you’re starting the hormone injections or whatever, but we should be doing that with every single person that’s been established on it; in fact probably more so.”* |
|  | EPs are confident they can support exercise behaviour of men on ADT due to having knowledge and experience of delivering and adapting exercise for clinical populations. Initial apprehensions and questions were addressed during professional training and confidence continued to develop overtime. | **EPC1**: “I*t’s another bow to our string, but also especially with our background at Nuffield, with the clinical side that we deliver anyway we have the experience and it makes it more interesting as well.”*  **EPC1**: “*I think 50/50 because as well I think we were the first ones ever to do it for the country, so we stepped in and this thing, right how it was working, how it was trialling. I think the more we got involved in it and the more we tailored it so it helped them, the members as much as sort of, I think accomplish has grown.”*  **EPW1**: “*Yeah so I attended the same training as the PTs. So I’d had all the training that they’d had anyway. So that was always the intention because if the PTs are delivering anything, whether it’s group exercise classes, whether it’s particular programmes like STAMINA then the FM should always attend the training as well so that they have an understanding of what’s happening. So yeah I was fully prepared.”*  **EPW1**: “*I think any level 3 qualified personal trainers, I think what Nuffield were looking for was personal trainers with an established client base as well. Because then it’s a proven track record almost. You know that with the training then that those men should then be in safe hands with those PTs, so… The training did prepare us quite heavily for men who were resistant to change and quite, yeah resistant to exercise potentially. And a lot of the training was geared quite heavily around that message of how you have those conversations and you’re trying to change those mindsets and things. But we didn’t actually experience any of that when it came to it. All the men wanted to be here, so whether that’s an element in the training that perhaps didn’t need to be quite so heavily included.”*  **EPS1**: “*We are trained to do that for this reason. You know, we’re not just here just to deliver exercise to fit healthy people. That’s not the point of being a personal trainer. That’s not what we choose to do it for really. So yeah, I think that’s ideal really. We have all the knowledge of the exercises, and the adaptations and that sort of thing, so we can make it inventive and different without compromising on their health as well.”*  **EPS1**: “*I suppose there’s two different ways of thinking about it, so if I was still PTing a lot and I was part of that and I was getting involved in that for me personally it would be really great. Because I do know that I have clients that are getting older and potentially going through this sort of thing, so that’s really great. But it’s also just something I really would like to have the confidence in doing so that I know that I can help people in that way. You know, with my clients a lot more it is about helping them with what’s going on around them and what they’re going through, rather than helping them lose weight or that sort of thing. It is more about kind of just keeping them ticking them over with where they are and helping them through their situation.”*  **EPS1**: “*With my particular role I felt OK, because that’s something that I do every day in terms of talking to someone around the gym, and working out who they are and what their personality is, and what support they will need. But, yeah, in terms of my team having gone to the training I felt confident that I had a team that knew what they were doing and they felt comfortable. But they’d also come and talk to me if they need anything, so yeah.”*  **EPC/S2**: “*Yeah, to be honest not really a problem; we’re quite confident with it. I know from the PTs when I was speaking them a little bit, I know they were quite unsure, and it was something they were a bit apprehensive about, which is why I think they’ve come to me quite a bit with questions etc. But I think the training was key in addressing a lot of that for those individuals. But yeah myself quite confident, and it was a good training initially from XXX giving me a bit more of a background into the different aspects and things to consider with the specific medication that the individuals are on.”*  **EPC3**: “*It was all very clear and insightful really about what we’re going to be doing….. When it came to actually getting into the gym, like when STAMINA started, I was a bit out of the loop with it as in it just kind of happened. One minute’s it was like oh STAMINA clients are here. And I don’t know whether that was again the gym or whether it was the STAMINA project, so it was more of a what do we do when they’re here, because the training and when they came was a bit further apart, so maybe a little refresher from management here or somebody coming in and saying right this is how it’s going to work, you’re going to have your sheets, you’re going to do this and that. And things like that, I suppose, just kind of refreshing before it comes in. So I guess yeah knowing how everything’s going to work, what we need to do with them, things like that. So I was a bit kind of clueless I guess when they got here with the paperwork and stuff. So I had to like, I think that was why I rang up the first time actually, it was, it was about can we split it half and half, cardio and half weights and things like that - because as far as I was aware it was strict on the fact that it has to be 45 and 15, but then they said if they want to do 30/30, that’s fine, just go for it, so it’s kind of knowing what we can and can’t do, I suppose. So, but again I suppose that’s just like a refresher needed.”*  **EPC3**: “*Again it was more that not knowing what to expect. Are we going to hurt them, are we going to overwork them or are we going to underwork them, it was that not knowing. But I guess you get that with anyone. So yeah confident enough to get going, because I know how to train people, but more so how they’re going to be yeah, because sometimes you actually see them when they’re in and you’ll know who they are, sometimes you don’t. So you just kind of get told you’ve got somebody and then that’s it, you’re kind of, you’re not knowing what you’re going to expect. You can read their notes and stuff, but like I say sometimes it can sound like they’re going to actually fall to pieces if they do certain things. So whether like an introduction would be nice first. I suppose, yeah.”* |
